# Clinical Utility of Circulating Biomarkers for Diagnosis and Prognosis of Acute Stroke in the Emergency Department: A Narrative Review from Current Evidence to Clinical Implementation

**DOI:** 10.3390/diagnostics16183053

**Published:** 2026-09-20

**Authors:** Mihaela Cristina Marin, George Țocu, Lavinia Țocu, Loredana Stavăr Matei, Ionica Grigore, Bogdan Ioan Ștefănescu, Sorin Ion Berbece

**Affiliations:** 1 Faculty of Medicine and Pharmacy, Research Center in the Medical-Pharmaceutical Field, “Dunărea de Jos” University, 800008 Galati, Romania; cristina.marin@ugal.ro (M.C.M.); lavinia.tocu@ugal.ro (L.Ț.); ionica.grigore@ugal.ro (I.G.); bogdan.stefanescu@ugal.ro (B.I.Ș.); sorin.berbece@ugal.ro (S.I.B.); 2 “Sf. Ioan” Children’s Emergency Hospital, 800487 Galati, Romania; 3 “Sf. Apostol Andrei” County Emergency Clinical Hospital, 800578 Galati, Romania

**Keywords:** acute stroke, emergency department, biomarkers, diagnosis, prognosis

## Abstract

Acute stroke requires rapid diagnostic classification and prognostic assessment, yet clinical examination and neuroimaging do not fully characterize cerebral injury. This narrative review evaluated the diagnostic, prognostic, and pathophysiological relevance of circulating biomarkers in acute stroke, emphasizing emergency department applications. PubMed, Scopus, Web of Science, Embase, and Google Scholar were searched from January 2015 to August 2026, supplemented by landmark studies. Evidence on brain-specific and systemic biomarkers was synthesized narratively, including multimarker panels, point-of-care testing, and artificial intelligence-based models. Glial fibrillary acidic protein has shown consistent evidence for early differentiation of intracerebral hemorrhage from ischemic stroke. Combinations of glial fibrillary acidic protein and D-dimer showed potential for large-vessel occlusion detection and prehospital triage. Neurofilament light chain, S100B, neuron-specific enolase, matrix metalloproteinase-9, inflammatory indices, hemostatic markers, cardiac biomarkers, and metabolic signatures were associated with severity, neurological deterioration, hemorrhagic transformation, mortality, functional outcome, or recurrence. Clinical translation remains limited by heterogeneity in populations, sampling times, analytical platforms, thresholds, and external validation. Circulating biomarkers are most likely to be useful as adjuncts to established stroke assessment. Multimarker strategies, point-of-care platforms, and integration with clinical scales, neuroimaging, and artificial intelligence may require standardization, multicenter validation, and demonstration of additional clinical value.

## 1. Introduction

Stroke is a major neurological emergency and remains an important cause of mortality, long-term disability, cognitive impairment, and loss of functional independence [1,2]. The acute phase is particularly critical because neurological injury evolves rapidly and diagnostic and therapeutic decisions must often be made within a limited time window [3]. The Emergency Department (ED) therefore represents a key point in the stroke pathway, where initial stabilization, neurological assessment, laboratory testing, neuroimaging, and therapeutic eligibility are established [3].

Acute stroke includes ischemic and hemorrhagic events with distinct pathophysiological mechanisms and management requirements [3,4]. Rapid differentiation is essential because therapeutic strategies differ substantially, particularly when reperfusion treatment is considered [3,4]. Diagnostic uncertainty is further increased by neurological, metabolic, cardiovascular, infectious, toxic, and functional conditions that may present with stroke-like manifestations [5].

Current evaluation integrates clinical history, neurological examination, standardized severity scales, routine laboratory testing, and neuroimaging [3]. Non-contrast computed tomography is central for the detection of intracranial hemorrhage, while computed tomography angiography, perfusion imaging, and magnetic resonance imaging provide additional information on vascular occlusion, cerebral perfusion, infarct extent, and tissue viability [3,6]. Although neuroimaging remains essential, availability, acquisition time, organizational constraints, and patient-related factors may affect the speed and completeness of emergency assessment [6].

Routine laboratory investigations contribute information on metabolic abnormalities, hematological and coagulation status, organ function, systemic inflammation, and cardiovascular injury [3,4]. However, these parameters are not specific to cerebral tissue damage and cannot independently determine the presence, subtype, or extent of an acute cerebrovascular event [7]. This limitation has stimulated interest in circulating biomarkers reflecting neuronal and glial injury, endothelial dysfunction, blood–brain barrier disruption, inflammation, thrombosis, oxidative stress, and metabolic disturbance [7].

Stroke biomarkers comprise brain-derived and systemic molecules originating from neural tissue, vascular structures, circulating cells, inflammatory and coagulation pathways, and organs affected by the acute event [7,8]. Brain-specific proteins may reflect neuronal, astrocytic, axonal, or myelin injury, whereas inflammatory, hematological, hemostatic, endothelial, cardiac, and metabolic biomarkers characterize complementary components of the biological response to stroke [8,9,10]. More recent research has expanded toward microRNAs, extracellular vesicles, proteomic and metabolomic signatures, and other molecular patterns identified through omics-based technologies [11,12].

The interpretation of circulating biomarkers is influenced by stroke subtype, lesion burden, time from symptom onset, sampling interval, analytical methodology, reperfusion treatment, and associated comorbidities [8,12]. These factors contribute to substantial heterogeneity among published studies and complicate direct comparison between individual biomarkers [8]. Clinical translation therefore depends not only on biological plausibility, but also on analytical robustness and performance within the time-sensitive environment of acute stroke care.

This narrative review adopts an emergency-care-centered perspective on circulating biomarkers in acute stroke, encompassing both acute ischemic stroke and spontaneous intracerebral hemorrhage. It evaluates their pathophysiological basis, diagnostic and prognostic utility, and potential clinical integration, with particular attention to differentiation from stroke mimics, ischemic–hemorrhagic discrimination, large-vessel occlusion triage, and early prognostic stratification. Biomarker specificity, multimarker strategies, point-of-care testing, and integration with clinical scales, neuroimaging, and artificial intelligence are also considered, together with the analytical and translational limitations that currently influence their readiness for incorporation into acute stroke pathways.

## 2. Materials and Methods

This article was designed as a narrative review of the literature on circulating biomarkers in acute stroke, with particular emphasis on their diagnostic and prognostic relevance in the ED. The objectives were to summarize the pathophysiological basis of major biomarker categories and evaluate their reported applications in stroke diagnosis, subtype differentiation, large-vessel occlusion detection, severity assessment, outcome prediction, and emergency clinical decision-making. Multimarker panels, composite bioscores, point-of-care testing, and integration with clinical scales, neuroimaging, and AI-based models were also considered.

A literature search was conducted in PubMed/MEDLINE, Scopus, Web of Science, and Embase. Google Scholar was used as a supplementary source for additional publications and citation tracking. The search covered January 2015 to August 2026, while earlier landmark studies were included when relevant to the biological basis, initial validation, or historical development of specific biomarkers.

Search terms included combinations of “acute stroke biomarkers,” “ischemic stroke biomarkers,” “intracerebral hemorrhage biomarkers,” “stroke mimic biomarkers,” “large-vessel occlusion biomarkers,” “stroke prognosis biomarkers,” and “point-of-care stroke biomarkers.” Additional targeted searches were performed for specific biomarker classes and individual biomarkers discussed in the review. Boolean operators “AND” and “OR” were adapted to the requirements of each database. Reference lists of relevant original studies, systematic reviews, meta-analyses, and clinical guidelines were also screened manually.

Eligible publications included peer-reviewed studies involving adults with suspected or confirmed acute stroke and evaluating circulating biomarkers in relation to diagnosis, differentiation between ischemic and hemorrhagic stroke, distinction from stroke mimics, large-vessel occlusion, stroke severity, neurological deterioration, hemorrhagic transformation, mortality, functional outcome, or recurrent vascular events. Original prospective and retrospective studies and diagnostic accuracy studies were prioritized, while systematic reviews, meta-analyses, consensus documents, and clinical guidelines were used primarily to support background, pathophysiological, and methodological considerations.

The review primarily focused on acute ischemic stroke and spontaneous intracerebral hemorrhage, which represent the main cerebrovascular entities addressed in the available literature on circulating biomarkers for emergency diagnosis and prognostic stratification. Subarachnoid hemorrhage was considered for clinical context as part of the spectrum of hemorrhagic stroke but was not a principal focus of the biomarker analysis.

Studies focused exclusively on chronic stroke, rehabilitation, pediatric populations, experimental animal models, or in vitro research without direct clinical relevance were excluded. Conference abstracts without sufficient data, editorials, and non-peer-reviewed publications were excluded. Case reports were not used as primary evidence for stroke biomarker performance. Only English-language publications were considered.

Titles and abstracts were reviewed for relevance, followed by full-text evaluation of potentially relevant publications. Articles were selected narratively according to their relevance to the objectives of the review, considering study design, population characteristics, sample size, biomarker type, timing of blood collection, analytical method, comparator or reference standard, and reported diagnostic or prognostic outcomes. Particular attention was given to prospective studies, multicenter cohorts, independent validation, and clinically applicable sampling intervals.

Extracted information included study design, sample size, stroke subtype, clinical setting, biological specimen, sampling time, analytical platform, comparator group, diagnostic or prognostic endpoint, and principal findings. Diagnostic studies were evaluated with attention to sensitivity, specificity, discrimination, and proposed decision thresholds, whereas prognostic studies were considered in relation to outcome definitions, adjustment for confounders, and validation strategy. No formal risk-of-bias tool or evidence-grading system was applied because the article was designed as a narrative rather than a systematic review.

Because of substantial heterogeneity in biomarkers, analytical methods, sampling intervals, study populations, diagnostic definitions, and outcomes, quantitative meta-analysis was not performed. Evidence was synthesized narratively according to biomarker category, pathophysiological mechanism, diagnostic application, prognostic relevance, and potential integration into acute stroke pathways.

## 3. Acute Stroke in the Emergency Department: Clinical and Diagnostic Context

The ED is the setting in which suspected acute stroke must be rapidly recognized, differentiated from alternative diagnoses, and classified according to its major cerebrovascular subtype [3,5]. Because clinical presentation alone is often insufficient for definitive diagnosis, particularly during the hyperacute phase, evaluation relies on the integration of neurological findings, laboratory data, and neuroimaging [3,5]. Clinicians must also determine therapeutic eligibility and identify patients at risk of early deterioration [3]. These diagnostic and prognostic uncertainties motivate the evaluation of circulating biomarkers as potential adjuncts to acute stroke assessment.

### 3.1. Acute Ischemic and Hemorrhagic Stroke

Acute stroke is broadly divided into ischemic and hemorrhagic forms, which differ substantially in vascular mechanisms, patterns of cerebral injury, and immediate management [13,14]. Acute ischemic stroke results from thrombotic or embolic arterial occlusion, leading to reduced cerebral perfusion and progressive tissue ischemia [13]. The extent of injury depends on the vascular territory involved, duration of occlusion, collateral circulation, and residual blood flow [15]. Large-vessel occlusion is particularly relevant in emergency care because rapid recognition may determine eligibility for endovascular reperfusion therapy. In the randomized MR CLEAN trial, intra-arterial treatment significantly improved functional outcomes in patients with acute ischemic stroke caused by proximal intracranial arterial occlusion [16].

Hemorrhagic stroke results from rupture of an intracranial vessel and includes intracerebral hemorrhage and subarachnoid hemorrhage. Intracerebral hemorrhage, which represents the principal hemorrhagic subtype considered in this review, causes direct tissue disruption, hematoma formation, mass effect, and secondary injury related to edema, inflammation, and blood degradation products [14]. Subarachnoid hemorrhage involves bleeding into the subarachnoid space and is most commonly associated with rupture of an intracranial aneurysm, often presenting with sudden severe headache and meningeal symptoms [17]. Although subarachnoid hemorrhage is briefly described here as part of the clinical spectrum of hemorrhagic stroke, it is not a primary focus of the subsequent biomarker analysis.

Clinical manifestations overlap substantially between stroke subtypes. Focal motor or sensory deficits, aphasia, visual disturbances, impaired coordination, and altered consciousness may occur in both ischemic and hemorrhagic events. Headache, vomiting, seizures, or severe hypertension may increase suspicion of hemorrhage but are not sufficiently specific for definitive classification [13,14,17].

Emergency differentiation between ischemic and hemorrhagic stroke therefore remains dependent on neuroimaging [13]. Their distinct biological patterns of neuronal injury, glial activation, coagulation, inflammation, endothelial dysfunction, and blood–brain barrier damage provide the rationale for investigating subtype-specific circulating biomarkers [7,8]. These biological differences may also influence the timing, magnitude, and relative predominance of circulating biomarker release during the hyperacute and early acute phases of stroke.

### 3.2. Stroke Mimics and Diagnostic Uncertainty

Stroke mimics are nonvascular conditions that produce acute neurological symptoms resembling ischemic or hemorrhagic stroke [5]. Common causes include seizures with postictal deficits, migraine with aura, hypoglycemia, functional neurological disorders, peripheral vestibular syndromes, toxic or metabolic encephalopathies, central nervous system infections, and intracranial tumors [5]. In a study of 2167 consecutive patients transported to the ED with suspected stroke, 35.2% were ultimately diagnosed with a stroke mimic, with seizures and infections among the most frequent etiologies [18].

Several factors complicate initial differentiation between true stroke and mimicking conditions. Neurological deficits may be transient, fluctuating, or atypical, while the medical history may be incomplete because of aphasia, impaired consciousness, cognitive dysfunction, or absence of witnesses. Posterior circulation stroke is particularly challenging because vertigo, gait instability, diplopia, dysarthria, nausea, and impaired coordination may overlap with nonvascular vestibular or neurological disorders. In one study, 37% of posterior circulation strokes were initially misdiagnosed, compared with 16% of anterior circulation strokes [19]. Small or very early ischemic lesions may also remain inconspicuous on initial non-contrast computed tomography [20].

Certain features may favor an alternative diagnosis, including gradual symptom progression in migraine, witnessed seizure activity, rapid reversal after correction of hypoglycemia, or examination findings inconsistent with recognized neuroanatomical patterns [5]. Clinical studies of patients presenting with suspected stroke have nevertheless shown that such features do not reliably exclude an acute cerebrovascular event [21]. Stroke may also coexist with metabolic disturbances, seizures, infection, or other systemic conditions, further increasing diagnostic uncertainty [5].

Misclassification has important clinical consequences: delayed recognition of true stroke may postpone time-sensitive treatment, whereas stroke mimics may undergo unnecessary investigations or treatment. In the Get With The Guidelines–Stroke Registry, 2517 stroke mimics were identified among 72,582 patients treated with intravenous thrombolysis for presumed ischemic stroke, representing 3.5% of treated cases [22]. Initial assessment therefore requires integration of neurological examination, blood glucose, routine laboratory testing, symptom evolution, vascular risk factors, and neuroimaging [5]. This persistent diagnostic overlap represents an important setting in which circulating biomarkers may complement established emergency assessment.

### 3.3. Current Clinical, Laboratory, and Imaging Assessment

Current assessment of suspected acute stroke relies on the rapid integration of clinical examination, routine laboratory testing, and neuroimaging. Initial evaluation establishes symptom onset or last known well time, neurological severity, previous functional status, vascular risk factors, medication use, and potential contraindications to acute treatment [23]. Neurological impairment is commonly quantified using the National Institutes of Health Stroke Scale (NIHSS), although clinically significant posterior circulation or strategically located lesions may present with relatively low scores. In a study of 1569 patients with acute ischemic stroke, posterior circulation strokes had substantially lower baseline NIHSS scores than anterior circulation strokes, and 15% of patients with posterior circulation stroke and NIHSS scores ≤ 4 still had an unfavorable 3-month outcome [24].

Routine laboratory testing supports differential diagnosis and therapeutic decision-making and generally includes blood glucose, complete blood count with platelet count, electrolytes, renal function, and coagulation parameters [23]. Cardiac troponin and electrocardiography are also frequently assessed because cardiovascular abnormalities are common in acute stroke [23]. In a cohort of 1067 patients with acute ischemic stroke undergoing serial high-sensitivity cardiac troponin measurements, acute myocardial injury was identified in 25.3% and chronic myocardial injury in 40.4% [25]. Additional laboratory investigations may be selected according to the clinical context. Although essential for detecting metabolic abnormalities, bleeding risk, organ dysfunction, and associated conditions, routine laboratory tests are not sufficiently specific to confirm acute cerebral injury [7].

Neuroimaging remains the cornerstone of stroke classification. Non-contrast computed tomography is primarily used to detect intracranial hemorrhage, major structural abnormalities, and early signs of extensive ischemia [6]. Computed tomography angiography identifies vascular occlusion and other arterial abnormalities, while perfusion imaging may characterize ischemic core and hypoperfused tissue [6]. Magnetic resonance imaging, particularly diffusion-weighted imaging, provides high sensitivity for acute ischemic lesions, including small and posterior circulation infarctions [6]. In a prospective comparison of 356 patients with suspected acute stroke, magnetic resonance imaging showed a sensitivity of 83% compared with 26% for computed tomography for the diagnosis of any acute stroke, with the greatest difference observed for acute ischemic lesions [26].

Despite this multimodal approach, early ischemic abnormalities may remain subtle, advanced imaging is not universally available, and routine laboratory parameters largely reflect systemic rather than brain-specific processes [6,7]. These limitations support the investigation of circulating biomarkers as complementary components of acute stroke assessment.

### 3.4. Unmet Diagnostic and Prognostic Needs in Emergency Care

Despite established stroke pathways, important diagnostic and prognostic uncertainties remain during the first hours of emergency evaluation [19,26]. Cerebral ischemia may be difficult to confirm when symptoms are mild, atypical, fluctuating, or accompanied by initially inconclusive imaging findings [20,24,26]. Diagnostic uncertainty is particularly relevant in posterior circulation events and in patients with stroke-like manifestations caused by nonvascular conditions [5,19].

Early identification of vascular and tissue characteristics that influence treatment decisions also remains challenging. Prehospital clinical severity scales show variable and generally moderate discrimination for large-vessel occlusion, and no single scale provides uniformly accurate classification. In the prospective PRESTO study of 1039 patients with suspected stroke, eight prehospital stroke scales achieved areas under the curve ranging from 0.72 to 0.83 for anterior-circulation large-vessel occlusion, compared with 0.86 for the clinician-assessed NIHSS [27]. Advanced vascular and perfusion imaging provides essential information, but access is not uniform and interhospital transfer may delay definitive treatment. In the prospective STRATIS registry, transferred patients had a median onset-to-revascularization time of 311.5 min compared with 202.0 min for patients presenting directly to an endovascular-capable center and showed lower rates of functional independence [28].

Prognostic assessment is similarly complex. During the hyperacute phase, clinicians must estimate the risk of neurological deterioration, cerebral edema, hematoma expansion, hemorrhagic transformation, treatment-related complications, mortality, and long-term disability [13,14]. In a prospective multicenter cohort of 9114 patients with acute ischemic stroke, early neurological deterioration occurred in 14.1% of patients, with 62.5% of events developing within the first 24 h and a significant association with poor 90-day outcome [29]. Clinical scales and neuroimaging provide important prognostic information but cannot fully characterize the biological heterogeneity and dynamic systemic response associated with acute cerebral injury.

Routine laboratory parameters contribute to emergency assessment but generally lack sufficient specificity for cerebral tissue injury, stroke subtype, or individual outcome prediction [10]. This has sustained interest in biomarkers capable of providing additional information within a clinically relevant timeframe.

An ideal biomarker for emergency stroke care should be rapidly measurable, analytically reproducible, minimally influenced by common comorbidities, and capable of adding clinically relevant information beyond established diagnostic methods. Its role would be to complement, rather than replace, neurological assessment and neuroimaging by improving diagnostic confidence, risk stratification, and early decision-making.

## 4. Pathophysiological Basis of Stroke Biomarkers

Circulating biomarkers in acute stroke arise from multiple biological processes triggered by cerebral ischemia or intracranial hemorrhage [30,31]. These include neuronal and glial injury, blood–brain barrier disruption, inflammatory and endothelial activation, coagulation abnormalities, oxidative stress, and metabolic dysfunction [30,31]. Understanding these mechanisms is essential for interpreting the origin, temporal profile, and potential clinical significance of biomarkers measured during the acute phase.

### 4.1. Neuronal and Glial Injury and Blood–Brain Barrier Disruption

Neuronal and glial injury is a major source of circulating biomarkers in acute stroke [32]. In ischemic stroke, reduced cerebral blood flow limits oxygen and glucose delivery, causing energy failure, membrane depolarization, ionic imbalance, excitotoxicity, and progressive cellular damage [32]. Neurons within the ischemic core undergo rapid irreversible injury, whereas surrounding hypoperfused tissue may remain temporarily viable [33]. The extent of damage depends on ischemia duration and severity, collateral circulation, vascular territory, and timing of reperfusion [33].

Glial cells are also directly affected [34]. Astrocytes contribute to metabolic support, neurotransmitter regulation, ion homeostasis, and maintenance of the neurovascular unit; ischemia may induce astrocytic activation, swelling, structural disruption, and release of intracellular proteins [34]. Oligodendrocyte injury contributes to myelin and axonal damage, while microglial activation initiates inflammatory signaling after tissue injury [34].

In hemorrhagic stroke, neural injury results initially from mechanical tissue disruption and hematoma formation [14]. Compression, impaired local perfusion, and exposure to thrombin, hemoglobin, heme, iron, and other blood degradation products contribute to secondary neuronal and glial injury [14]. In a prospective magnetic resonance imaging study of intracerebral hemorrhage, progressive erythrolysis within the hematoma was demonstrated, and perihematomal iron accumulation was associated with erythrolysis and hematoma volume [35].

Blood–brain barrier disruption is closely linked to these processes [36]. Endothelial injury, degradation of intercellular junctions and extracellular matrix components, inflammation, and oxidative stress increase vascular permeability, promoting cerebral edema and facilitating entry of brain-derived molecules into the systemic circulation [36]. In a human dynamic contrast-enhanced magnetic resonance imaging study, blood–brain barrier permeability was significantly greater within infarcted tissue than in homologous contralateral regions, with the largest relative increase observed between 6 and 48 h after acute ischemic stroke [37].

Proteins derived from neurons, astrocytes, axons, and myelin can consequently become detectable in peripheral blood after acute cerebral injury [31,36]. Their concentrations depend on lesion size, cellular origin, barrier permeability, molecular properties, clearance mechanisms, and sampling time [31]. These mechanisms provide the biological basis for the investigation of brain-specific circulating biomarkers in acute stroke.

### 4.2. Neuroinflammation, Endothelial Dysfunction, and Thrombotic Mechanisms

Neuroinflammation develops rapidly after both ischemic and hemorrhagic stroke and contributes to secondary cerebral injury [38]. Damaged neural cells activate microglia, astrocytes, endothelial cells, and circulating immune cells, promoting release of cytokines, chemokines, reactive oxygen species, and adhesion molecules and subsequent recruitment of leukocytes into injured tissue [38].

Inflammatory activation is closely linked to endothelial dysfunction [39]. The cerebral endothelium normally regulates vascular tone, blood–brain barrier integrity, leukocyte trafficking, platelet activity, coagulation, and fibrinolysis [39]. During acute stroke, ischemia, oxidative stress, inflammatory mediators, and altered shear forces impair these functions, promoting reduced nitric oxide bioavailability, adhesion molecule expression, platelet activation, leukocyte adhesion, and increased vascular permeability [39]. In a prospective cohort of 52 patients with acute ischemic stroke undergoing endovascular thrombectomy, post-reperfusion concentrations of interleukin-6, vascular cell adhesion molecule-1, and intercellular adhesion molecule-1 increased progressively, supporting dynamic inflammatory and endothelial activation after reperfusion [40].

Thrombotic mechanisms in ischemic stroke involve coordinated interactions among platelets, coagulation factors, endothelial cells, and the vascular wall [41]. Exposure of tissue factor and subendothelial structures promotes platelet adhesion and thrombin generation, followed by fibrin formation and thrombus stabilization [41,42]. In cardioembolic stroke, thrombi typically originate within the heart, whereas large-artery atherosclerotic stroke is commonly associated with plaque disruption and local thrombosis [41].

Inflammation and thrombosis are further connected through immunothrombotic pathways [42]. Activated leukocytes can enhance coagulation, while neutrophil extracellular traps provide a scaffold for platelet and fibrin accumulation [42]. In a study of thrombi retrieved from 68 patients with acute ischemic stroke, neutrophils and neutrophil extracellular traps were detected in nearly all analyzed specimens [43]. Impairment of endogenous anticoagulant and fibrinolytic pathways may further promote thrombus persistence and microvascular obstruction [42].

In hemorrhagic stroke, coagulation is necessary to limit bleeding, but excessive thrombin activity and inflammatory signaling may contribute to perihematomal edema and secondary tissue injury [14,38]. These interacting inflammatory, endothelial, and hemostatic mechanisms provide the biological basis for investigating circulating cytokines, adhesion molecules, coagulation factors, fibrinolytic products, and endothelial biomarkers in acute stroke.

### 4.3. Oxidative Stress and Metabolic Dysfunction

Oxidative stress and metabolic dysfunction are closely interconnected mechanisms of secondary cerebral injury in acute stroke [44,45]. The brain is particularly vulnerable because of its high energy requirements, dependence on continuous oxygen and glucose delivery, and limited energy reserves [44]. Interruption of cerebral perfusion rapidly impairs mitochondrial oxidative phosphorylation, reduces adenosine triphosphate production, and disrupts cellular ion homeostasis [44].

In ischemic stroke, energy failure promotes membrane depolarization, intracellular calcium accumulation, glutamate excitotoxicity, and activation of enzymes that damage proteins, lipids, and nucleic acids [44]. Mitochondrial dysfunction, activated leukocytes, enzymatic pathways, and impaired antioxidant defenses further increase reactive oxygen species production, contributing to lipid peroxidation, endothelial dysfunction, and membrane injury [44,45]. In a study comparing 100 patients with acute ischemic stroke with 100 age- and sex-matched healthy controls, malondialdehyde concentrations were significantly higher and total antioxidant power significantly lower in the stroke group [46].

Reperfusion restores oxygen and nutrient delivery but may transiently intensify oxidative injury through rapid reoxygenation of ischemic tissue [45]. Endogenous antioxidant systems, including superoxide dismutase, catalase, and glutathione peroxidase, normally limit oxidative damage, although their protective capacity may become insufficient during severe cerebral injury [47].

Metabolic abnormalities extend beyond cellular energy failure. Anaerobic glycolysis increases lactate production and tissue acidosis, while disturbances in glucose metabolism may influence neuronal survival, edema, and vascular function [44]. Hyperglycemia may aggravate oxidative and inflammatory responses, whereas hypoglycemia can produce stroke-like deficits and further compromise cerebral energy metabolism [44]. In the prospective multicenter STAY ALIVE registry of 695 patients undergoing recanalization therapy, admission hyperglycemia was independently associated with poor 90-day functional outcome, mortality, and symptomatic intracranial hemorrhage [48].

In hemorrhagic stroke, erythrocyte degradation and release of hemoglobin, heme, and free iron enhance oxidative stress and contribute to membrane damage, mitochondrial dysfunction, and perihematomal injury [35,49]. Enzymatic and metabolic sources may further amplify oxidative stress; NADPH oxidase 2 (NOX2) generates superoxide and participates in redox-dependent inflammatory signaling [50], while dopamine oxidation represents an additional mechanism capable of generating reactive oxygen species and quinone intermediates [51].

These processes generate circulating markers of lipid peroxidation, oxidative DNA damage, antioxidant activity, glucose metabolism, lactate accumulation, and other metabolic disturbances [46,47]. Their concentrations vary according to stroke subtype, lesion severity, systemic metabolic status, treatment, and sampling time [47].

**Figure 1:**
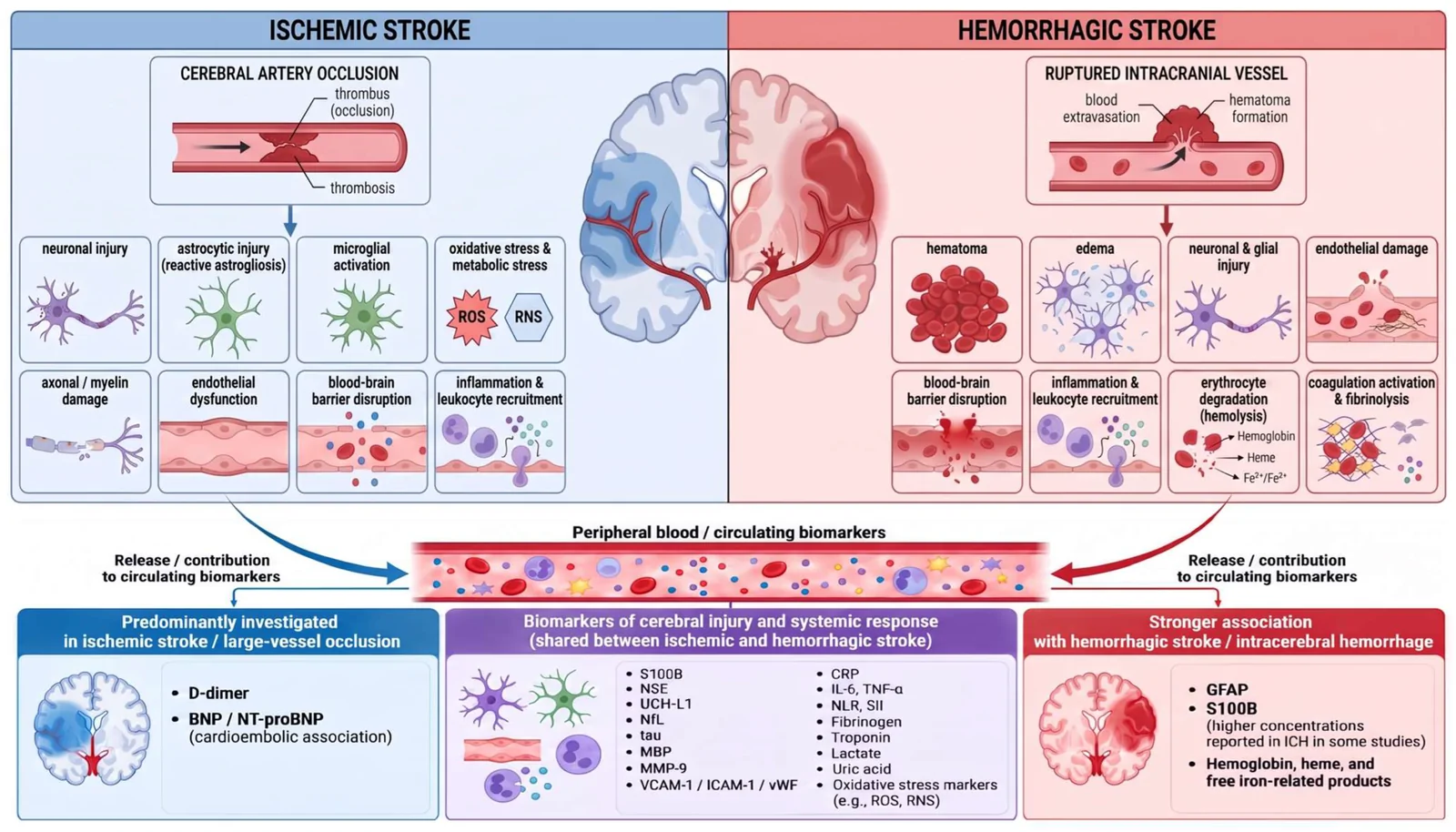
Pathophysiological origin and systemic release of major circulating biomarkers in acute stroke. Acute ischemic stroke is initiated by cerebral artery occlusion and thrombosis, leading to neuronal and astrocytic injury, microglial activation, axonal and myelin damage, endothelial dysfunction, blood–brain barrier disruption, inflammation and leukocyte recruitment, and oxidative and metabolic stress. Hemorrhagic stroke results from rupture of an intracranial vessel with blood extravasation and hematoma formation and is accompanied by edema, neuronal and glial injury, endothelial damage, blood–brain barrier disruption, inflammation and leukocyte recruitment, erythrocyte degradation, and activation of coagulation and fibrinolysis. These interconnected processes contribute to the release or modulation of brain-derived and systemic biomarkers detectable in the peripheral circulation. The lower panel groups biomarkers according to their predominant reported associations. D-dimer and BNP/NT-proBNP have been particularly investigated in ischemic stroke, including large-vessel occlusion and cardioembolic mechanisms. Most neuronal, axonal, neurovascular, inflammatory, hemostatic, cardiac, and metabolic biomarkers reflect biological processes shared by ischemic and hemorrhagic stroke. GFAP shows a stronger association with intracerebral hemorrhage and has been investigated for early differentiation from ischemic stroke, while higher S100B concentrations have also been reported in intracerebral hemorrhage in some studies. Hemoglobin, heme, and free iron-related products reflect erythrocyte degradation and secondary injury after intracerebral hemorrhage. The associations illustrated are predominant rather than exclusive and should not be interpreted as absolute stroke-subtype specificity. Abbreviations: BNP, B-type natriuretic peptide; CRP, C-reactive protein; GFAP, glial fibrillary acidic protein; ICAM-1, intercellular adhesion molecule 1; ICH, intracerebral hemorrhage; IL-6, interleukin 6; MBP, myelin basic protein; MMP-9, matrix metalloproteinase 9; NfL, neurofilament light chain; NLR, neutrophil-to-lymphocyte ratio; NSE, neuron-specific enolase; NT-proBNP, N-terminal pro-B-type natriuretic peptide; RNS, reactive nitrogen species; ROS, reactive oxygen species; SII, systemic immune–inflammation index; TNF-α, tumor necrosis factor alpha; UCH-L1, ubiquitin C-terminal hydrolase L1; VCAM-1, vascular cell adhesion molecule 1; vWF, von Willebrand factor.

The principal cellular and vascular mechanisms contributing to the systemic release of circulating biomarkers in acute ischemic and hemorrhagic stroke are summarized in Figure 1.

## 5. Brain-Specific and Neurovascular Biomarkers

Brain-specific and neurovascular biomarkers are of particular interest in acute stroke because they originate from cellular and structural components directly involved in cerebral injury [52]. Their circulating concentrations may reflect damage to neurons, astrocytes, axons, myelin, the extracellular matrix, and the neurovascular unit, but are influenced by lesion characteristics, blood–brain barrier permeability, and sampling time [31,53]. Major candidates include glial fibrillary acidic protein, S100 calcium-binding protein B, neuron-specific enolase, ubiquitin C-terminal hydrolase L1, neurofilament proteins, tau proteins, and markers of vascular and extracellular matrix injury [52,53].

An important limitation of these biomarkers is that they primarily reflect the type and extent of neural or glial injury rather than the specific etiology of that injury. Increased circulating concentrations of NfL, GFAP, tau, S100B, and related proteins have also been reported in other neurological disorders characterized by neuroaxonal or astroglial damage, including neurodegenerative and neuroinflammatory diseases [52,53]. Accordingly, these biomarkers should be interpreted as indicators of cerebral or neuroaxonal injury whose diagnostic value in acute stroke depends on the clinical context, temporal profile, and integration with neuroimaging and other diagnostic information.

### 5.1. Glial Fibrillary Acidic Protein and S100 Calcium-Binding Protein B

Glial fibrillary acidic protein (GFAP) and S100 calcium-binding protein B (S100B) are among the most extensively investigated astrocyte-associated biomarkers in acute cerebral injury [52,53]. Both may enter the systemic circulation following astrocytic injury, particularly when blood–brain barrier disruption facilitates their passage from cerebral tissue into blood [36,52]. Their biological characteristics and temporal release patterns differ, influencing their potential diagnostic and prognostic applications [52,53].

GFAP is an intermediate filament protein predominantly expressed in astrocytes and involved in cytoskeletal stability [52]. Acute astrocytic injury can increase circulating concentrations, with particularly rapid release after intracerebral hemorrhage because of direct mechanical tissue disruption and hematoma formation [52,53]. In ischemic stroke, circulating GFAP generally rises more gradually and is influenced by infarct extent and sampling time [52,53]. In a prospective multicenter study of 205 patients with suspected acute stroke, plasma GFAP concentrations were markedly higher in intracerebral hemorrhage than in ischemic stroke, with an area under the curve of 0.915 and 84.2% sensitivity and 96.3% specificity at a cutoff of 0.29 μg/L [54]. In a subsequent prospective multicenter validation study of 202 patients, GFAP again showed significantly higher concentrations in intracerebral hemorrhage, with 77.8% sensitivity and 94.2% specificity at a cutoff of 0.03 μg/L; concentrations also correlated positively with hemorrhage volume [55].

These findings have supported GFAP as a candidate for early differentiation between intracerebral hemorrhage and ischemic stroke. Its interpretation nevertheless depends on assay sensitivity, sampling interval, lesion characteristics, and the presence of other neurological conditions associated with astrocytic injury [52,53]. Increased concentrations may also occur in traumatic brain injury, neurodegenerative disorders, and other structural cerebral diseases [52].

S100B is a calcium-binding protein predominantly expressed by astrocytes but is not exclusively brain-specific [56]. It participates in calcium regulation, cellular metabolism, and neurotrophic signaling, while cerebral injury and increased barrier permeability can increase its circulating concentration [36,56]. In a prospective study of 332 patients with acute ischemic stroke, S100B and interleukin-6 were the only circulating biomarkers independently associated with infarct volume after adjustment for stroke etiology [57].

The clinical specificity of S100B is limited by extracerebral expression, including adipose tissue and skeletal muscle, and its concentration may also be influenced by trauma, systemic conditions, and renal clearance [52,56]. Moreover, S100B often rises progressively after cerebral injury, reducing its usefulness as an isolated hyperacute marker [52,56]. In a prospective study of 1072 patients with acute ischemic stroke treated with intravenous thrombolysis, higher S100B concentrations at 24 h were independently associated with larger infarct volume, greater neurological severity, hemorrhagic transformation, and unfavorable 3-month functional outcome [58].

GFAP and S100B therefore provide complementary information on astrocytic and neurovascular injury. GFAP is generally more brain-specific, whereas S100B is more susceptible to extracerebral influences [52,56]. Their clinical utility depends on temporal kinetics, analytical methodology, and integration with clinical and imaging findings.

### 5.2. Neuron-Specific Enolase, Ubiquitin C-Terminal Hydrolase L1, and Neurofilament Light Chain

Neuron-specific enolase (NSE), ubiquitin C-terminal hydrolase L1 (UCH-L1), and neurofilament light chain (NfL) reflect different components of neuronal and axonal injury [52]. Their circulating concentrations depend on the extent of structural damage, blood–brain barrier permeability, molecular kinetics, and sampling time [52].

NSE is a glycolytic enzyme predominantly expressed in neurons and neuroendocrine cells [52]. Neuronal injury promotes its release into extracellular fluid and subsequently into the circulation. Its clinical interpretation is limited by incomplete brain specificity, delayed kinetics, and susceptibility to preanalytical interference, particularly hemolysis because erythrocytes contain substantial amounts of NSE. In a prospective study of 79 patients with acute ischemic stroke undergoing reperfusion therapy, serum NSE concentrations at 48 h were significantly higher in patients with unfavorable 90-day outcomes, and levels above 26.3 ng/mL were independently associated with a 13.5-fold higher risk of unfavorable functional outcome [59].

UCH-L1 is a neuronal cytoplasmic enzyme involved in the ubiquitin–proteasome system and intracellular protein turnover [52]. Its release after structural brain injury may occur relatively early and primarily reflects neuronal cell-body damage, although increased concentrations are not specific to stroke. In a clinical study of 177 patients presenting with acute stroke or transient ischemic attack, serum UCH-L1 concentrations were significantly elevated in intracerebral hemorrhage compared with healthy controls, but the marker did not reliably distinguish intracerebral hemorrhage from ischemic stroke [60].

NfL is a structural component of the neuronal cytoskeleton that is particularly abundant in myelinated axons [52]. Axonal disruption results in its release into extracellular fluid and eventually into blood, where sensitive immunoassays permit measurement at low concentrations. In two independent ischemic stroke cohorts, serial serum NfL concentrations increased from admission, peaked around day 7, correlated with infarct volume, and independently predicted 3-month functional outcome [61]. Its relatively slow kinetics limit its usefulness for hyperacute diagnosis but make it relevant as a marker of evolving axonal injury. In a prospective cohort of 595 patients with ischemic stroke, NfL concentrations varied according to sampling time and were associated with stroke severity and poor short- and long-term outcomes [62].

NSE, UCH-L1, and NfL therefore provide complementary information on neuronal cytoplasmic injury, neuronal cell-body damage, and axonal disruption, respectively. Their clinical interpretation requires consideration of sampling time, analytical methodology, and extracerebral or chronic neurological factors that may influence circulating concentrations.

### 5.3. Tau, Myelin-Associated, and Other Neuronal Biomarkers

Tau proteins and myelin-associated molecules provide complementary information on neuronal, axonal, and white-matter injury in acute stroke [52,53]. These biomarkers reflect structural damage involving microtubules, axons, and myelinated fibers rather than exclusively neuronal cell-body injury.

Tau is a microtubule-associated protein involved in stabilization of the axonal cytoskeleton and intracellular transport [52]. Disruption of neuronal and axonal integrity can promote its release into cerebrospinal fluid and blood. An early clinical study identified increased serum tau as a marker of axonal damage in acute ischemic stroke [63]. More recently, brain-derived tau has been investigated as a potentially more specific measure of central nervous system-derived tau. In two independent hospital-based cohorts comprising 713 patients with ischemic stroke, plasma brain-derived tau showed a strong correlation with cerebral infarct volume (ρ = 0.72) [64]. In the prospective PROMISE study of 502 patients with acute ischemic stroke, brain-derived tau increased during the first 24 h, while changes from admission to day 2 correlated with infarct progression and day-2 concentrations were strongly associated with final infarct volume [65].

The temporal kinetics of tau remain important for clinical interpretation because circulating concentrations may increase as neuronal and axonal injury evolves rather than immediately after stroke onset [52,53]. Age and pre-existing neurodegenerative disease may also affect baseline concentrations, limiting the specificity of isolated measurements.

Myelin basic protein (MBP) is a major structural component of the myelin sheath, and its release reflects white-matter and myelinated axonal injury [52]. Its relatively delayed appearance in blood limits its potential for hyperacute diagnosis. In an analysis of 359 patients from the NINDS recombinant tissue plasminogen activator Stroke Study, higher peak MBP concentrations were associated with greater baseline neurological severity and larger lesion volumes, while smaller increases during the first 24 h were observed in patients with favorable outcomes [66]. Evidence from other neurological disorders further illustrates the limited disease specificity of neuroaxonal injury biomarkers. In Wilson’s disease, increased serum NfL has been associated with neurological involvement, disease severity, and neuroimaging evidence of brain injury [67].

Other neuronal candidates associated with synaptic integrity, cytoskeletal disruption, and neuronal metabolism have also been investigated [52,53]. Their interpretation remains limited by incomplete specificity for acute cerebrovascular injury and by potential influences from chronic neurological disease, systemic conditions, analytical methodology, and sampling time. Tau, MBP, and related biomarkers may therefore complement other brain-derived markers by reflecting axonal degeneration, cytoskeletal disruption, and myelin injury.

### 5.4. Matrix Metalloproteinases and Neurovascular Injury Biomarkers

Matrix metalloproteinases are zinc-dependent proteolytic enzymes involved in extracellular matrix remodeling and vascular integrity [68]. In acute stroke, their activation is associated with endothelial injury, neurovascular unit disruption, and increased blood–brain barrier permeability [36,68]. Matrix metalloproteinase-9 (MMP-9) has received particular attention because of its ability to degrade basement membrane and tight-junction-associated structures [68].

In ischemic stroke, MMP-9 expression may increase in response to inflammatory signaling, oxidative stress, leukocyte activation, and reperfusion [45,68]. Excessive proteolytic activity promotes extracellular matrix degradation, vascular leakage, and vasogenic edema and may contribute to hemorrhagic transformation [36,68]. In a prospective study of 250 patients with hemispheric ischemic stroke, admission plasma MMP-9 concentrations were markedly higher in patients who subsequently developed hemorrhagic transformation, and levels ≥ 140 ng/mL were independently associated with this complication, with an odds ratio of 12 [69]. In another study of patients undergoing endovascular thrombectomy, those who developed hemorrhagic transformation showed a significantly greater increase in arterial MMP-9 concentrations immediately after the procedure [70].

Matrix metalloproteinases are also involved in intracerebral hemorrhage [71]. Inflammatory activation and exposure to blood degradation products stimulate proteolytic pathways within perihematomal tissue, contributing to extracellular matrix degradation, edema, microvascular instability, and secondary neurovascular injury [71]. In a prospective study of 57 patients with spontaneous intracerebral hemorrhage, increased plasma MMP-9 concentrations were associated with perihematomal edema volume and neurological worsening in patients with deep hemorrhage [72]. Matrix metalloproteinase activity is nevertheless time-dependent, as later phases may also contribute to tissue remodeling, angiogenesis, and recovery [68,71].

Other circulating markers of neurovascular injury include molecules reflecting endothelial activation and vascular dysfunction, such as vascular cell adhesion molecule-1, intercellular adhesion molecule-1, thrombomodulin, and von Willebrand factor [39]. In a prospective study of 64 patients with acute ischemic stroke treated with thrombolysis, lower concentrations of soluble thrombomodulin and soluble endothelial protein C receptor were independently associated with successful arterial recanalization [73].

The clinical interpretation of neurovascular biomarkers is limited by incomplete cerebral specificity because systemic inflammation, cardiovascular disease, infection, malignancy, and other vascular disorders may alter their circulating concentrations [39,68]. Their potential value therefore depends on stroke subtype, sampling time, clinical context, and integration with brain-derived biomarkers and neuroimaging.

### 5.5. Emerging Brain-Specific Biomarkers

Advances in molecular profiling and high-sensitivity analytical technologies have expanded the range of candidate biomarkers investigated in acute stroke beyond conventional neuronal and glial proteins [12,53]. Emerging approaches increasingly focus on molecular signatures that reflect specific cellular compartments, mechanisms of cerebral injury, and intercellular communication.

Circulating microRNAs are small non-coding RNA molecules that regulate gene expression and can originate from neurons, glial cells, endothelial cells, and circulating immune cells [12]. Their expression may change in response to neuronal injury, inflammation, endothelial dysfunction, angiogenesis, and tissue remodeling, while their relative stability in blood supports their investigation as circulating biomarkers [12]. Analytical variability related to sample processing, normalization, and platform selection remains an important limitation [12,53]. In a study including 191 patients with acute ischemic stroke and 61 with transient ischemic attack, a panel of 11 circulating microRNAs was consistently differentially regulated between the two groups across discovery and validation cohorts [74].

Extracellular vesicles represent another emerging source of molecular information [11]. These membrane-bound particles transport proteins, lipids, messenger RNA, and microRNAs and may originate from neurons, astrocytes, microglia, oligodendrocytes, endothelial cells, or circulating blood cells [11]. In a clinical study of 47 patients with acute ischemic stroke, platelet-derived extracellular vesicles measured at admission were associated with initial stroke severity and mid-term outcome, while T-cell-derived extracellular vesicles were also related to early and 3-month outcomes [75]. Cell-specific extracellular vesicle profiling may therefore provide information on the cellular origin and molecular characteristics of cerebral injury.

Circulating cell-free nucleic acids are also being investigated as indicators of cellular damage. In a prospective cohort of 92 patients with large-vessel occlusion undergoing mechanical thrombectomy, higher cell-free DNA concentrations were associated with unfavorable outcome, while 7-day concentrations were independently associated with mortality [76].

Proteomic and metabolomic technologies further enable simultaneous assessment of large numbers of molecules and identification of multidimensional signatures associated with stroke phenotypes [12]. In an integrated untargeted metabolomic and proteomic study using discovery and independent validation cohorts, a combined model incorporating one metabolite and three proteins achieved an area under the curve of 0.985 in the discovery set [77].

The principal challenge for these emerging biomarkers is translation from exploratory molecular studies to standardized clinical testing [12,53]. Analytical complexity, biological variability, limited external validation, and absence of universally accepted decision thresholds currently restrict routine implementation. Nevertheless, these approaches extend stroke biomarker research from individual circulating proteins toward multidimensional molecular profiles capable of capturing several components of cerebral injury simultaneously.

## 6. Inflammatory, Hemostatic, Cardiac, and Metabolic Biomarkers

Systemic biomarkers complement brain-specific markers by reflecting inflammatory activation, coagulation disturbances, cardiovascular stress, and metabolic alterations associated with acute stroke [7,10]. Although most lack cerebral specificity, they are readily measurable in peripheral blood and may provide clinically relevant information when interpreted together with neurological findings, neuroimaging, and stroke subtype [10].

### 6.1. C-Reactive Protein, Cytokines, and Inflammatory Mediators

Inflammatory activation occurs rapidly after acute cerebral injury and involves both local neuroinflammatory mechanisms and a systemic acute-phase response [38]. C-reactive protein (CRP) is among the most widely investigated circulating inflammatory biomarkers in acute stroke because it is readily available, inexpensive, and routinely measured in clinical laboratories [52]. Its hepatic synthesis is largely driven by interleukin-6 (IL-6) and other proinflammatory signals, while circulating concentrations primarily reflect systemic inflammatory activity rather than cerebral injury alone [38].

The interpretation of CRP and related inflammatory biomarkers is influenced by sampling time and by numerous extracerebral conditions, including cardiovascular disease, obesity, infection, malignancy, and chronic inflammatory disorders. The broader clinical literature illustrates the substantial inflammatory burden that may accompany hospital-acquired infections [78], severe intra-abdominal inflammatory and infectious conditions [79], and complex clinical settings combining infection, immunodeficiency, and malignancy [80]. These factors are relevant when interpreting inflammatory biomarkers in patients presenting with acute neurological symptoms because they may contribute independently to elevated circulating inflammatory markers.

High-sensitivity C-reactive protein (hsCRP) assays permit measurement of lower circulating concentrations and have been investigated in relation to vascular inflammation and cerebrovascular outcomes. In a prospective study of 3653 patients with first-ever ischemic stroke, higher hsCRP concentrations were associated with neurological deterioration and poor 3-month functional outcome [81].

Cytokines provide more direct information on inflammatory signaling after stroke [38]. IL-6, interleukin-1 beta (IL-1β), and tumor necrosis factor alpha (TNF-α) are among the principal proinflammatory mediators involved in interactions between microglia, astrocytes, endothelial cells, and circulating leukocytes, whereas interleukin-10 (IL-10) contributes to counter-regulatory anti-inflammatory responses [38]. Their circulating concentrations may vary according to lesion burden, stroke subtype, reperfusion treatment, infection, and sampling interval. In a prespecified substudy of the Third China National Stroke Registry involving 10,472 patients with acute ischemic stroke or transient ischemic attack, higher IL-6 concentrations were independently associated with recurrent stroke and poor functional outcome at 1 year [82].

Chemokines and related inflammatory mediators further contribute to leukocyte recruitment and communication between injured cerebral tissue and the systemic immune compartment [38]. Their interpretation remains challenging because neuroinflammatory and systemic inflammatory responses are not specific to stroke and may also occur in other neurological and systemic disorders. Consequently, altered concentrations of CRP, cytokines, and related inflammatory mediators should be regarded as indicators of inflammatory activity rather than stroke-specific biomarkers when interpreted in isolation. The limited specificity of CRP and IL-6 is further illustrated by their diagnostic and prognostic associations in severe systemic inflammatory conditions such as sepsis [83]. Inflammatory and cytokine responses are also observed across other neurological disorders, including neurodegenerative and neuroinflammatory diseases, further limiting the disease specificity of these biomarkers [84]. Therefore, in acute stroke, these inflammatory biomarkers are most informative when interpreted together with clinical findings, neuroimaging, sampling time, and potential extracerebral sources of inflammation.

### 6.2. Hematological Ratios and Cellular Inflammatory Indices

Routine complete blood count parameters provide indirect information on the systemic inflammatory and immune response accompanying acute stroke. Derived hematological ratios are particularly attractive because they integrate changes in different circulating cell populations while remaining rapidly available from routine laboratory data.

The neutrophil-to-lymphocyte ratio (NLR) is the most extensively investigated of these indices. Acute stroke is frequently accompanied by neutrophilia and relative lymphopenia, reflecting activation of innate immunity together with stress-related changes in adaptive immune function [38]. NLR therefore provides a simple measure of the balance between neutrophil-driven inflammation and lymphocyte-mediated immune regulation. Its interpretation may be influenced by stroke severity, infection, comorbidities, treatment, and sampling time.

The platelet-to-lymphocyte ratio (PLR) integrates platelet and lymphocyte counts and reflects interactions between thrombosis and inflammation. Platelets contribute to thrombus formation as well as endothelial activation and inflammatory signaling [42]. PLR may therefore provide complementary information on thromboinflammatory activity, although hematological disorders, antiplatelet therapy, systemic inflammation, and other factors affecting platelet or lymphocyte counts may influence its values. In 741 patients with acute ischemic stroke treated with intravenous thrombolysis, higher PLR measured 24 h after treatment was independently associated with poor 3-month functional outcome and mortality, whereas admission PLR was not [85].

More complex indices combine several cellular components of the inflammatory response. The systemic immune–inflammation index (SII) incorporates platelet, neutrophil, and lymphocyte counts. In a prospective cohort of 697 patients with acute ischemic stroke, higher SII was associated with an increased risk of early neurological deterioration, particularly in large-artery atherosclerotic stroke [86]. The systemic inflammation response index (SIRI), which combines neutrophil, monocyte, and lymphocyte counts, provides an additional measure of systemic immune activation. In 861 patients with acute ischemic stroke, NLR, PLR, and SIRI were independently associated with poor functional outcome at discharge, whereas the lymphocyte-to-monocyte ratio showed an inverse association [87]. A subsequent prospective five-center cohort of 1011 patients with ischemic stroke or transient ischemic attack further showed that higher baseline SII and SIRI were associated with poor outcomes and that longitudinal changes in inflammatory status provided additional prognostic information [88].

The main advantages of hematological ratios are their low cost, rapid availability, and compatibility with routine emergency laboratory testing. However, they remain nonspecific and may be affected by infection, malignancy, corticosteroid therapy, chronic inflammatory disease, physiological stress, and hematological abnormalities. Their interpretation should therefore remain integrated with the broader clinical and laboratory context.

### 6.3. D-Dimer, Fibrinogen, and Other Hemostatic Biomarkers

Hemostatic biomarkers reflect coagulation activation, fibrin formation, platelet activity, and fibrinolysis, processes closely involved in both ischemic and hemorrhagic stroke [41,89]. D-dimer and fibrinogen are among the most frequently investigated because they are widely available in clinical laboratories and provide complementary information on thrombus formation and degradation [89].

D-dimer is a fibrin degradation product generated during plasmin-mediated breakdown of cross-linked fibrin and therefore reflects activation of both coagulation and fibrinolysis [89]. In acute ischemic stroke, increased concentrations may be associated with greater thrombotic burden, cardioembolic mechanisms, large-vessel occlusion, or systemic prothrombotic conditions. In the prospective multicenter Stroke-Chip study including 1308 patients with suspected acute stroke, D-dimer was independently associated with large-vessel occlusion, which was identified in 262 patients [90]. In another study of 98 consecutive patients with first-ever acute ischemic stroke, D-dimer concentrations were significantly higher in cardioembolic stroke than in other etiological subtypes and remained independently associated with cardioembolic etiology after multivariable analysis [91]. Its specificity is nevertheless limited because increased concentrations may also occur with infection, venous thromboembolism, malignancy, trauma, pregnancy, and advanced age [89].

Fibrinogen is a soluble plasma glycoprotein converted to fibrin by thrombin and also contributes to platelet aggregation and blood viscosity [41,89]. As both a coagulation factor and an acute-phase reactant, increased concentrations may reflect prothrombotic activity and systemic inflammation [89]. Fibrinogen levels may also be influenced by vascular risk factors, smoking, metabolic disease, inflammatory status, and sampling time. In a prospective cohort of 1851 patients with acute ischemic stroke, a nonlinear relationship was observed between fibrinogen concentration and 3-month functional outcome, with levels above 2.74 g/L associated with an increased risk of unfavorable outcome [92].

Other hemostatic biomarkers include von Willebrand factor, factor VIII, tissue factor, thrombin–antithrombin complexes, fibrin degradation products, plasminogen activator inhibitor-1, antithrombin, and proteins C and S [42,73,89]. These markers reflect different components of endothelial activation, thrombin generation, clot formation, and fibrinolytic regulation [42,89]. In hemorrhagic stroke, routine hemostatic assessment is particularly important because coagulation abnormalities and anticoagulant exposure may influence ongoing bleeding and hematoma expansion.

Hemostatic biomarkers primarily reflect systemic rather than exclusively cerebral processes. Their clinical interpretation should therefore consider stroke subtype, medication exposure, inflammatory status, associated diseases, and neuroimaging findings.

### 6.4. Cardiac Biomarkers

Cardiac biomarkers are frequently altered during acute stroke and may reflect pre-existing cardiovascular disease, a cardioembolic mechanism, or acute cardiac injury triggered by the cerebrovascular event [93]. Their interpretation is clinically relevant because cardiac disorders and stroke share multiple risk factors and may directly influence stroke etiology and early clinical evolution.

Cardiac troponins are the principal biochemical markers of myocardial injury [93]. Increased concentrations in acute stroke may result from acute coronary syndrome, chronic structural heart disease, renal dysfunction, or stroke-associated myocardial injury [25,93]. Severe cerebral injury may activate sympathetic and neuroendocrine pathways, leading to catecholamine excess, myocardial stress, microvascular dysfunction, and cardiomyocyte injury within the spectrum of stroke–heart syndrome [93]. In the prospective TRELAS cohort of 1016 patients with acute ischemic stroke, elevated high-sensitivity cardiac troponin T was present in more than half of patients; even moderate elevations were associated with unfavorable functional outcome, while dynamic changes were associated with increased in-hospital mortality [94].

Troponin concentrations should be interpreted together with their temporal dynamics, as serial measurements can help distinguish acute myocardial injury from chronically elevated values [25,94]. Electrocardiographic findings, echocardiography, clinical symptoms, renal function, and previous cardiovascular history provide additional diagnostic context. The limited specificity of troponin elevation is also illustrated by its occurrence in other acute cardiac and systemic conditions [95].

B-type natriuretic peptide and N-terminal pro-B-type natriuretic peptide are released in response to myocardial wall stress and increased intracardiac pressure and have been investigated in relation to atrial fibrillation, cardiac dysfunction, and cardioembolic stroke [10]. In a prospective study of 279 patients with acute ischemic stroke, N-terminal pro-B-type natriuretic peptide concentrations were significantly higher in cardioembolic than in non-cardioembolic stroke; a cutoff of 332 pg/mL provided 98.3% sensitivity and 75.8% specificity for identifying cardioembolic etiology [96]. Interpretation remains influenced by age, renal function, heart failure, structural cardiac disease, and rhythm abnormalities [10].

In this context, cardiac biomarkers may also help identify patients requiring more detailed cardiovascular evaluation, particularly when stroke etiology remains uncertain or when occult atrial fibrillation or concomitant myocardial injury is suspected [10,93].

Other cardiac-related biomarkers, including creatine kinase myocardial band, copeptin, and markers of myocardial stress or fibrosis, have also been explored, although their role in acute stroke remains less established [10]. Cardiac biomarkers are therefore most informative when integrated with electrocardiography, cardiac imaging, rhythm monitoring, neuroimaging, and the broader clinical context.

### 6.5. Metabolic, Oxidative Stress, and Omics-Derived Biomarkers

Metabolic and oxidative stress biomarkers reflect disturbances in energy production, redox balance, and systemic metabolic regulation accompanying acute cerebral injury [44,45]. Most are not specific to the central nervous system but may provide complementary information on stroke severity and the systemic response to tissue injury.

Blood glucose is routinely assessed in acute stroke. Hyperglycemia may reflect diabetes mellitus or the acute stress response and is associated with metabolic, oxidative, endothelial, and inflammatory disturbances [44]. Registry data have also linked diabetes and admission hyperglycemia with clinical outcomes after recanalization therapies for acute ischemic stroke [48]. Conversely, hypoglycemia may produce neurological deficits that mimic stroke and requires immediate recognition [5]. Lactate reflects anaerobic metabolism and tissue hypoxia, although its concentration is influenced by numerous extracerebral factors. In a study of 400 patients with cerebrovascular disease, admission hyperlactatemia (>2 mmol/L) was associated with significantly higher mortality at 1, 3, and 12 months [97].

Oxidative stress can be assessed through products of lipid, protein, and nucleic acid oxidation and through components of endogenous antioxidant defense [46,47]. Investigated markers include malondialdehyde, F2-isoprostanes, oxidized proteins, 8-hydroxy-2′-deoxyguanosine, uric acid, glutathione, superoxide dismutase, and catalase [46,47]. Their interpretation is complicated by stroke subtype, reperfusion status, metabolic comorbidities, renal and hepatic function, medication use, and sampling time [47].

Omics-based technologies enable simultaneous assessment of large numbers of biological molecules and support the development of multidimensional molecular signatures [12]. Circulating microRNA profiling has identified distinct expression patterns in acute ischemic stroke and transient ischemic attack [74]. Similarly, integrated metabolomic and proteomic analyses have identified combinations of circulating molecules associated with ischemic stroke, illustrating the potential of multi-omics approaches for biomarker discovery [77]. These strategies may capture biological processes related to neuronal injury, inflammation, coagulation, vascular dysfunction, and metabolic disturbance [12].

Differences in analytical platforms, data processing, patient populations, and validation strategies currently limit comparison across omics studies and their translation into routine clinical practice [12,53].

Representative clinical evidence for major circulating biomarkers in acute stroke is summarized in Table 1, while a broader comparative overview of their biological origin, temporal characteristics, potential clinical applications, and principal translational limitations is provided in Supplementary Table S1.

## 7. Diagnostic Utility of Biomarkers in Acute Stroke

The diagnostic value of circulating biomarkers in acute stroke depends on their ability to provide clinically relevant information during the earliest stages of evaluation [52,53]. Potential applications include distinguishing stroke from stroke mimics, differentiating ischemic from hemorrhagic stroke, identifying large-vessel occlusion, estimating lesion severity, and supporting prehospital and emergency department triage [52,53]. Their clinical usefulness depends not only on diagnostic accuracy but also on biomarker kinetics, analytical turnaround time, biological specificity, and additional value beyond established clinical and imaging methods.

### 7.1. Differentiation Between Stroke and Stroke Mimics

Distinguishing acute stroke from stroke mimics is an important potential application of circulating biomarkers [52,53]. Stroke mimics include heterogeneous neurological and systemic conditions such as seizures, migraine, metabolic disturbances, functional neurological disorders, infections, and peripheral vestibular syndromes [5,18]. Because many can produce sudden focal neurological deficits, clinical assessment alone may not always provide sufficient diagnostic certainty during the hyperacute phase.

Brain-derived biomarkers have been investigated as indicators of structural cerebral injury that may help differentiate stroke from conditions without substantial neuronal or glial damage [52,53]. GFAP, S100B, NSE, UCH-L1, NfL, and tau-related proteins have all been evaluated, although their diagnostic performance depends on lesion size and location, blood–brain barrier permeability, biomarker kinetics, and sampling time [31,52,53]. Small ischemic lesions or very early presentations may produce circulating concentrations below clinically useful thresholds.

Systemic biomarkers generally provide lower specificity for acute cerebral injury [7,10]. Inflammatory indices, hemostatic markers, metabolic parameters, and cardiac biomarkers may be altered in stroke but also in numerous conditions that can mimic or accompany it [52,89,93]. Stroke mimics themselves are biologically heterogeneous; seizures may produce transient neuronal injury and biomarker release, whereas metabolic or functional disorders may generate substantially different biochemical profiles [5,18]. A single universal biomarker is therefore unlikely to distinguish all mimicking conditions reliably.

Multimarker approaches attempt to combine complementary biological signals with clinical assessment [52,53]. In a study including 915 patients with stroke and 90 stroke mimics, a panel incorporating caspase-3, D-dimer, soluble receptor for advanced glycation end products, chimerin, secretagogin, and MMP-9 provided discriminatory information, with caspase-3 and D-dimer emerging as the most informative combination [98]. Conversely, in the prospective multicenter Stroke-Chip study of 1308 patients with suspected stroke, none of the 21 evaluated biomarkers improved discrimination between stroke and stroke mimics beyond clinical variables, which alone achieved a predictive accuracy of 80.8% [99].

These contrasting findings illustrate the challenges of biomarker-based differential diagnosis. In the emergency department, their most plausible role is as an adjunct to clinical assessment and neuroimaging, particularly when the initial presentation remains diagnostically uncertain.

### 7.2. Differentiation Between Ischemic and Hemorrhagic Stroke

Rapid distinction between ischemic and hemorrhagic stroke is essential because the two entities require different therapeutic approaches, particularly when reperfusion treatment is considered [13,14]. Neuroimaging remains the reference method for subtype classification, while circulating biomarkers have been investigated as complementary tools for earlier differentiation when imaging is delayed or not immediately available [52,53].

Glial fibrillary acidic protein (GFAP) has attracted particular interest because astrocytic disruption is generally more abrupt in intracerebral hemorrhage than during the earliest phase of ischemic stroke [52,53]. Earlier clinical studies showed substantially higher early circulating GFAP concentrations in intracerebral hemorrhage than in cerebral ischemia, supporting its potential for subtype discrimination [54,55]. In an unselected cohort of 299 patients with suspected stroke and symptom duration below 4.5 h, GFAP identified intracranial hemorrhage with an area under the curve of 0.73; a cutoff of 5369 pg/mL provided 100% specificity and 25% sensitivity, whereas a lower cutoff of 838 pg/mL increased sensitivity to 50.0% with 95.1% specificity [100].

S100B has also been investigated, although its lower cerebral specificity and later release may limit its value during the hyperacute phase [53,56]. In a study including 46 patients with intracerebral hemorrhage and 71 with ischemic stroke, plasma S100B concentrations were significantly higher in intracerebral hemorrhage; a cutoff of 67 pg/mL yielded an area under the curve of 0.903, with 95.7% sensitivity and 70.4% specificity [101]. Studies of neuron-specific enolase, ubiquitin C-terminal hydrolase L1, neurofilament light chain, and tau-related proteins have shown that these markers primarily reflect neural injury and frequently overlap between ischemic and hemorrhagic stroke, with performance influenced by lesion severity and sampling time [53,59,60,61,62,63,64,65].

Hemostatic and inflammatory biomarkers may provide additional information because ischemic and hemorrhagic stroke differ in thrombosis, tissue disruption, inflammation, and coagulation activation [53,89]. However, D-dimer, fibrinogen, coagulation parameters, cytokines, and hematological indices lack sufficient subtype specificity when used individually.

Multimarker strategies combining brain-derived proteins with systemic biomarkers may improve discrimination by integrating complementary biological mechanisms. Their clinical translation remains limited by variability in analytical platforms, sampling intervals, decision thresholds, stroke severity, and comorbidities [31,53]. Circulating biomarkers should therefore remain adjuncts to neuroimaging, with their main potential being to support rapid subtype-oriented assessment and diagnostic confidence within established stroke pathways.

### 7.3. Identification of Large-Vessel Occlusion and Assessment of Lesion Severity

Large-vessel occlusion (LVO) is a major therapeutic target in acute ischemic stroke because early recognition determines access to endovascular treatment and may influence transport to a thrombectomy-capable center [16,28]. Although neurological scales provide indirect information on stroke severity, they do not reliably identify all patients with LVO [27]. Circulating biomarkers have therefore been investigated as complementary tools for detecting major vascular occlusion and estimating cerebral tissue injury.

D-dimer is among the most extensively investigated markers in this setting. Increased concentrations may reflect greater thrombotic burden and are particularly associated with cardioembolic and other thromboembolic mechanisms [89,90,91]. Its specificity remains limited because levels are influenced by age, malignancy, infection, venous thromboembolism, and systemic coagulation activation [89]. In a study using two independent cohorts of patients with suspected stroke, D-dimer and GFAP formed the optimal biomarker combination for LVO detection; when combined with the FAST-ED clinical scale, the model achieved 95% diagnostic accuracy, with 91% sensitivity and 96% specificity in the initial cohort [102]. Subsequent prospective validation showed that GFAP, D-dimer, and FAST-ED achieved 94% specificity and 71% sensitivity, increasing to 93% specificity and 81% sensitivity among patients presenting within 6 h of symptom onset [103].

Brain-specific proteins may contribute to LVO assessment by reflecting the extent of downstream cerebral injury. GFAP, NfL, NSE, S100B, and related markers have been associated with infarct volume and neurological severity, although their temporal kinetics differ substantially [57,58,59,61]. In a study comparing large- and small-vessel ischemic stroke, serum GFAP and UCH-L1 concentrations were substantially higher in LVO, with median GFAP concentrations of 321.3 versus 58.6 pg/mL and UCH-L1 concentrations of 573.1 versus 251.8 pg/mL, respectively [104]. Early measurements may nevertheless remain low despite severe arterial occlusion, whereas later concentrations may more closely reflect established tissue injury [52,61].

Markers of neurovascular injury and blood–brain barrier disruption, including MMP-9, may provide additional information on lesion evolution, edema, and vascular permeability [68,69,70]. In hemorrhagic stroke, circulating biomarkers may similarly reflect hematoma volume, tissue disruption, and secondary injury [55,71,72].

Biomarker-based assessment of lesion severity is therefore influenced by anatomical extent, sampling time, reperfusion status, and secondary injury. Their most plausible role is within multimodal models combining circulating biomarkers with neurological severity scales and vascular or perfusion imaging.

### 7.4. Prehospital and Emergency Department Triage

Effective triage is essential in acute stroke because delays in recognition, transport, imaging, and treatment can directly affect therapeutic eligibility [28]. Prehospital assessment currently relies on symptom recognition, neurological examination, stroke severity scales, and transport protocols intended to identify patients requiring specialized stroke care [27,28]. However, atypical presentations, stroke mimics, posterior circulation events, and imperfect prediction of large-vessel occlusion (LVO) remain important limitations [19,27].

Prehospital stroke scales serve different clinical purposes. FAST and the Cincinnati Prehospital Stroke Scale (CPSS) are primarily used for rapid recognition of suspected stroke, whereas the Los Angeles Motor Scale (LAMS) incorporates motor deficit severity and has also been evaluated for identifying patients at increased likelihood of large-vessel occlusion. The National Institutes of Health Stroke Scale (NIHSS) provides a more detailed assessment of neurological deficit severity and is used predominantly in hospital-based stroke evaluation, although it may also be applied in specialized prehospital settings. In current prehospital practice, brief recognition and LVO-oriented scales may support initial triage and transport decisions, but none provides sufficiently accurate LVO classification to replace vascular imaging [23,27].

Circulating biomarkers have been investigated as potential adjuncts to prehospital and emergency department triage [100,103]. Clinically useful tests would need to provide rapid results with minimal sample processing and contribute information capable of influencing transport, imaging, or diagnostic decisions. Point-of-care platforms are particularly attractive because testing could be performed in ambulances, mobile stroke units, or immediately after emergency department arrival. In the prospective DETECT diagnostic accuracy study of 353 patients with suspected acute stroke, a 15-min point-of-care GFAP assay differentiated intracerebral hemorrhage from ischemic stroke and stroke mimics with an area under the curve of 0.880, while age-specific thresholds yielded sensitivities of 56.3–72.4% and specificities of 98.9–99.0% [105].

Brain-specific biomarkers such as GFAP may support early differentiation of hemorrhagic from ischemic stroke [54,55], whereas combinations incorporating D-dimer have been investigated for LVO detection [90,102,103]. Multimarker approaches may be particularly suitable for triage because they integrate complementary processes including astrocytic injury, thrombosis, neuronal damage, and vascular disruption. In a secondary analysis of prospectively collected samples from 210 patients with suspected stroke, a rapid point-of-care approach combining GFAP, D-dimer, and a clinical triage score achieved 75% sensitivity and 92% specificity for LVO detection and 99% specificity for intracerebral hemorrhage [106].

Biomarker-assisted triage could also support prioritization for advanced or vascular imaging and earlier transfer to thrombectomy-capable centers. In settings with limited specialist resources, rapid testing may help identify patients requiring accelerated referral.

At present, the clinical use of circulating biomarkers in acute stroke differs substantially between routine laboratory tests and stroke-specific biomarker assays. Conventional measurements such as blood glucose, coagulation parameters, cardiac troponin, and other routine laboratory tests are incorporated into acute stroke assessment primarily to identify treatment-relevant abnormalities, comorbidities, stroke mechanisms, or systemic complications rather than to establish the diagnosis of stroke itself [3]. In contrast, brain-derived biomarkers such as GFAP, NfL, S100B, and UCH-L1, as well as multimarker point-of-care approaches, remain predominantly investigational and are not currently established as routine stroke-specific diagnostic tests [31,53,105,106]. Broader implementation of these approaches requires short turnaround times, standardized analytical platforms, validated decision thresholds, consistent performance across stroke subtypes and time windows, and evidence that biomarker-guided testing improves clinical pathways beyond established neurological assessment and neuroimaging. Their most plausible current role is therefore as adjuncts to established prehospital scales, imaging strategies, and organized stroke networks.

**Figure 2:**
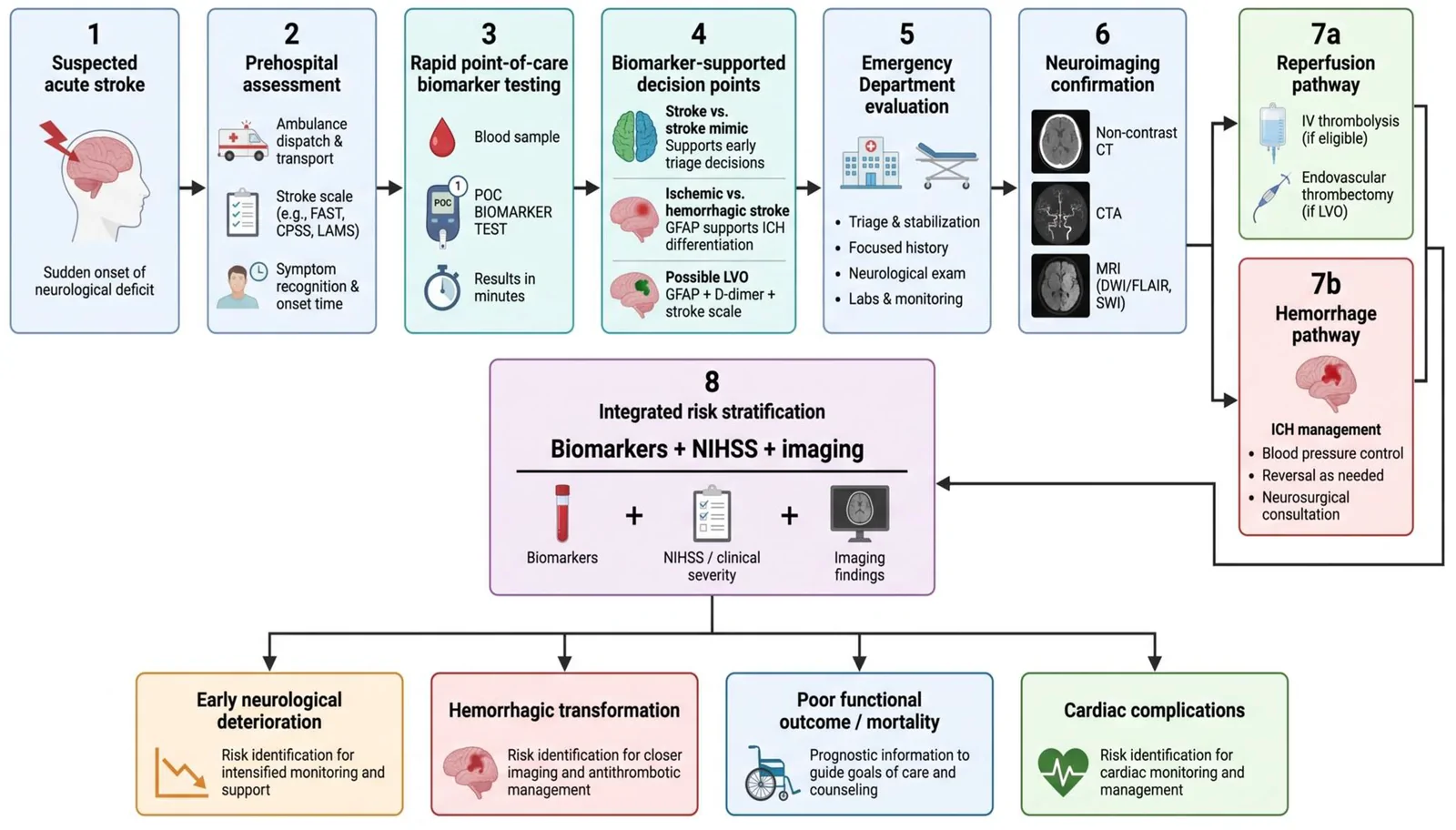
Potential Integration of Circulating Biomarkers into Prehospital and Emergency Department Stroke Pathways. Legend: The proposed workflow illustrates how rapid biomarker testing could complement established prehospital assessment, emergency department evaluation, and neuroimaging in patients with suspected acute stroke. Following initial clinical assessment, point-of-care biomarker testing may support early differentiation between stroke and stroke mimics, ischemic and hemorrhagic stroke, and possible large-vessel occlusion. Neuroimaging remains essential for diagnostic confirmation and guides subsequent reperfusion or hemorrhage-specific management. After the acute treatment pathway has been established, circulating biomarkers may be integrated with neurological severity and imaging findings for prognostic risk stratification, including early neurological deterioration, hemorrhagic transformation, poor functional outcome or mortality, and cardiac complications. Biomarker testing is intended as an adjunct to, rather than a replacement for, established clinical and imaging-based stroke assessment. Note: The structure and sequence of the workflow may vary according to institutional protocols, available resources, local stroke network organization, and access to point-of-care testing and advanced neuroimaging. Abbreviations: CPSS, Cincinnati Prehospital Stroke Scale; CT, computed tomography; CTA, computed tomography angiography; DWI, diffusion-weighted imaging; FAST, Face, Arm, Speech, Time; FLAIR, fluid-attenuated inversion recovery; GFAP, glial fibrillary acidic protein; ICH, intracerebral hemorrhage; IV, intravenous; LAMS, Los Angeles Motor Scale; LVO, large-vessel occlusion; MRI, magnetic resonance imaging; NIHSS, National Institutes of Health Stroke Scale; POC, point-of-care; SWI, susceptibility-weighted imaging.

The potential integration of circulating biomarkers into prehospital and emergency department stroke pathways, from initial triage to imaging confirmation, treatment selection, and subsequent risk stratification, is illustrated in Figure 2.

## 8. Prognostic Utility and Clinical Integration

Beyond diagnostic classification, circulating biomarkers are increasingly investigated for their ability to characterize the biological severity of acute stroke and anticipate early and long-term clinical evolution [9,52]. Potential prognostic applications include prediction of neurological deterioration, mortality, functional disability, hemorrhagic transformation, treatment-related complications, and recurrent vascular events [9,52]. Their clinical relevance depends on whether they provide additional information beyond established neurological scales and neuroimaging and whether they can be incorporated into reproducible multimarker or integrated prediction models.

### 8.1. Stroke Severity and Early Neurological Deterioration

Stroke severity reflects the extent and location of cerebral injury, vascular territory, collateral circulation, reperfusion, and secondary pathological processes [13,15]. Although the NIHSS remains the principal clinical tool for quantifying neurological impairment, circulating biomarkers may provide complementary information on tissue injury and biological responses not fully captured by clinical examination.

Brain-derived biomarkers have been investigated in relation to the magnitude of cerebral injury [52,53]. Higher concentrations of GFAP, S100B, NSE, NfL, tau-related proteins, and other neuronal or glial markers may accompany more extensive structural damage [57,58,59,61,64]. In a prospective cohort of 286 patients with acute ischemic stroke, higher serum GFAP concentrations were independently associated with greater neurological severity, including an increased likelihood of NIHSS scores > 6 [107]. These associations are influenced by sampling time because individual biomarkers have different release kinetics, and delayed increases may reflect evolving tissue damage more closely than neurological status at presentation [59,61,65].

Inflammatory and hematological markers may also provide information on stroke severity [86]. Increased inflammatory activity, reflected by CRP, cytokines, NLR, SII, and related indices, may accompany more extensive cerebral injury. In a study of 228 patients with first-ever anterior-circulation ischemic stroke, patients who developed early neurological deterioration had significantly higher NLR values than those without deterioration (median 3.8 vs. 2.4), and elevated NLR remained independently associated with deterioration after multivariable adjustment [108]. Hemostatic and endothelial biomarkers, including D-dimer, fibrinogen, MMP-9, and von Willebrand factor, may provide complementary information on thrombotic burden, vascular dysfunction, and blood–brain barrier injury [69,73,89,90,91,92].

Early neurological deterioration may result from infarct progression, persistent or recurrent arterial occlusion, cerebral edema, hemorrhagic transformation, hematoma expansion, seizures, or systemic complications [29]. In a prospective cohort of 319 patients with mild-to-moderate ischemic stroke, early neurological deterioration occurred in 27.9% of patients, and elevated serum NfL measured within 24 h was an independent predictor of deterioration [109]. Given the heterogeneity of these mechanisms, no single biomarker is likely to provide sufficient prognostic information across all patients.

Serial measurements may better capture evolving cerebral injury than a single baseline value. Integration of brain-specific, inflammatory, and hemostatic biomarkers with neurological severity and imaging findings may therefore provide a more comprehensive assessment of patients at risk of early deterioration.

### 8.2. Mortality and Functional Outcomes

Mortality and functional outcome are major prognostic endpoints after acute stroke and are influenced by neurological severity, lesion characteristics, age, comorbidities, stroke subtype, reperfusion status, and complications [9]. Circulating biomarkers may complement these factors by reflecting the extent of cerebral injury and the intensity of systemic biological responses.

Brain-derived biomarkers such as GFAP, S100B, NSE, NfL, and tau-related proteins have been associated with short- and long-term outcomes [58,59,61,64,65]. Higher concentrations generally indicate greater neuronal, glial, or axonal injury, although their prognostic relevance depends on sampling time and release kinetics [58,61,64,65]. In a prospective cohort of 213 patients with severe acute ischemic stroke, higher serum NfL and GFAP concentrations were independently associated with unfavorable 3-month functional outcome and improved the predictive performance of clinical models [110].

Inflammatory, hemostatic, endothelial, and cardiac biomarkers may provide complementary prognostic information by reflecting systemic inflammation, thrombosis, vascular dysfunction, and cardiovascular stress [73,86,87,88,92,93,94]. In a substudy of the Third China National Stroke Registry involving 10,518 patients with ischemic stroke or transient ischemic attack, higher baseline D-dimer concentrations were independently associated with all-cause mortality and poor functional outcome during 1-year follow-up [111]. In another cohort of 569 patients with ischemic stroke, elevated B-type natriuretic peptide was independently associated with lower odds of good functional outcome and higher mortality at 6 months, while also improving prognostic prediction in cardioembolic stroke [112].

Functional recovery remains influenced by rehabilitation, complications, baseline functional status, and broader clinical and social factors. Similarly, mortality may reflect not only cerebral injury but also age, organ dysfunction, infection, cardiovascular disease, and treatment response. Prognostic models integrating circulating biomarkers with neurological severity, neuroimaging, and relevant comorbidities are therefore likely to provide more clinically meaningful estimates than isolated biomarker thresholds.

### 8.3. Hemorrhagic Transformation, Treatment-Related Complications, and Recurrence

Hemorrhagic transformation is an important complication of acute ischemic stroke, ranging from petechial bleeding to clinically significant parenchymal hematoma [68,69]. Its development is influenced by infarct size, reperfusion, blood–brain barrier disruption, vascular fragility, hyperglycemia, anticoagulant exposure, and other clinical factors [48,68,69]. Biomarkers of endothelial injury, extracellular matrix degradation, coagulation, and inflammation have therefore been investigated for risk stratification [68,69,89].

MMP-9 has received particular attention because increased proteolytic activity may contribute to vascular basement membrane degradation and loss of blood–brain barrier integrity [68,69]. In a study of 327 patients with acute ischemic stroke treated with thrombolysis, an increase in circulating MMP-9 during the first 24 h was independently associated with symptomatic intracerebral hemorrhage and 3-month mortality [113]. Hemostatic parameters may provide complementary information on the balance between thrombosis and bleeding [89,92]. In a cohort of 1135 patients receiving reperfusion therapy, early fibrinogen depletion was independently associated with symptomatic intracranial hemorrhage after intravenous thrombolysis alone and after combined intravenous thrombolysis and endovascular thrombectomy [114]. Inflammatory indices may also reflect processes involved in vascular permeability and secondary tissue injury [86].

Biomarkers have also been evaluated for complications following intravenous thrombolysis or endovascular treatment, including intracranial hemorrhage, reperfusion injury, cerebral edema, and early neurological worsening [29,70,113,114]. Their interpretation remains influenced by initial stroke severity, treatment timing, recanalization success, and associated comorbidities.

Recurrent ischemic stroke and other vascular events represent a further prognostic concern [111]. Persistent thrombotic, inflammatory, cardioembolic, or endothelial abnormalities may be reflected by D-dimer, fibrinogen, inflammatory mediators, cardiac biomarkers, and endothelial markers [89,111]. In a cohort of 1431 patients with embolic stroke of undetermined source, 1-year recurrence increased progressively across D-dimer quartiles, with patients in the highest quartile having more than a sevenfold higher risk than those in the lowest quartile [115]. In a prospective study of 486 patients followed for more than 5 years, higher acute-phase concentrations of CD62E and macrophage migration inhibitory factor were independently associated with recurrent stroke or transient ischemic attack and improved prediction beyond vascular risk factors and CRP [116].

Because hemorrhagic transformation, treatment-related complications, and recurrence arise through different mechanisms, prognostic assessment is more appropriately based on combinations of biological markers with clinical variables, neuroimaging, stroke mechanism, and treatment-related factors.

### 8.4. Multimarker Panels and Composite Bioscores

The biological heterogeneity of acute stroke limits the prognostic value of individual biomarkers [9,52]. Neuronal injury, inflammation, endothelial dysfunction, thrombosis, cardiac stress, and metabolic disturbances evolve simultaneously but with different temporal profiles [30,38,44,52]. Multimarker panels aim to integrate these complementary biological signals and may therefore provide a broader representation of disease severity than isolated laboratory parameters. In a cohort of 3405 patients with ischemic stroke, the combined assessment of high-sensitivity CRP, complement C3, MMP-9, hepatocyte growth factor, and antiphosphatidylserine antibodies improved risk stratification for death or major disability at 3 months, with a net reclassification improvement of 28.5% when added to conventional risk factors [117].

Such panels may combine brain-specific, inflammatory, hematological, hemostatic, cardiac, and metabolic biomarkers. Their composition should be guided by biological plausibility and independent prognostic contribution rather than by simply increasing the number of laboratory variables.

Composite bioscores extend this concept by integrating several biomarkers, and potentially clinical or imaging variables, into a single numerical model. They may facilitate prediction of neurological deterioration, functional dependence, mortality, hemorrhagic transformation, and other complications. In a prospective study of 320 patients with ischemic stroke, 92 circulating biomarkers were evaluated and a Biomarker Panel Index was derived from 16 markers with the strongest independent associations. The index remained independently associated with 90-day mortality and increased the area under the receiver operating characteristic curve from 0.89 for the clinical model alone to 0.93 when incorporated into the prediction model [118].

Reliable bioscores require appropriate variable selection, control of collinearity and confounding, consideration of biomarker kinetics, and robust validation. Internal validation can assess model stability, but independent external validation remains essential before broader clinical application. In a study using a derivation cohort of 412 patients and an independent pooled validation cohort of 450 patients, copeptin, N-terminal pro-B-type natriuretic peptide, and mid-regional pro-atrial natriuretic peptide improved several measures of predictive performance beyond the Acute Stroke Registry and Analysis of Lausanne score in the derivation cohort, whereas external validation showed a more modest benefit [119].

Practical implementation is equally important. Multimarker models may show strong statistical performance but remain unsuitable for emergency use if they require numerous expensive assays or prolonged processing. Clinically useful panels should balance prognostic performance with analytical simplicity, rapid turnaround time, reproducibility, cost, and additional value beyond established clinical and imaging tools.

### 8.5. Integration with Clinical Scales, Neuroimaging, and Artificial Intelligence

The prognostic interpretation of circulating biomarkers is most relevant when integrated with established clinical and imaging parameters [120]. Clinical scales quantify neurological severity and functional status, while neuroimaging characterizes lesion burden, vascular occlusion, collateral circulation, edema, and other structural features [15,120]. Biomarkers may provide complementary information on tissue injury, inflammation, thrombosis, endothelial dysfunction, and systemic responses [117,118,119,120]. In a study of 103 patients with ischemic stroke, an integrated neural-network model combining clinical severity, carotid imaging, inflammatory, coagulation, and endothelial biomarkers predicted 1-year mortality with an area under the curve of 0.975 and a cross-validated accuracy of 93.3% [120].

The National Institutes of Health Stroke Scale and modified Rankin Scale remain central tools for assessing neurological severity and functional outcome [117,119]. Combining these measures with biomarkers and imaging parameters may improve prognostic stratification by integrating clinical, biological, and structural information [117,118,119,120]. In a multicenter registry of 4147 patients with acute ischemic stroke, a machine-learning model combining clinical variables with diffusion-weighted and apparent diffusion coefficient imaging achieved an area under the curve of 0.786 for unfavorable 3-month functional outcome, outperforming clinical or imaging models alone [121]. Similarly, in a study of 3338 patients, a magnetic resonance imaging-based deep-learning biomarker achieved an area under the curve of 0.788 and improved the performance of several established clinical risk scores [122].

AI and machine-learning approaches may further facilitate the integration of laboratory, clinical, imaging, and demographic data in acute stroke [120,121,122,123,124]. Potential applications include prediction of neurological deterioration, functional outcome, mortality, hemorrhagic transformation, treatment response, and recurrent vascular events [121,122,123,124]. Model performance nevertheless depends on dataset quality, representativeness, standardized biomarker sampling, and consistent analytical and imaging methods [123,124]. Broader experience from other complex clinical settings has similarly highlighted aspects of prognostic assessment, multimodal diagnostic evaluation, and individualized clinical decision-making [125,126,127].

Clinical implementation will require transparent model development, appropriate validation, calibration across independent populations, and comparison with established prognostic tools. Integrated approaches are likely to be most useful when they combine complementary biological, clinical, and imaging information while remaining interpretable, reproducible, and practical for routine stroke care.

## 9. Limitations

Several limitations of this review should be acknowledged. As a narrative review, study identification and selection may be subject to selection bias despite the literature search strategy. Only English-language publications were considered, which may have resulted in the omission of relevant evidence published in other languages. In addition, no formal risk-of-bias assessment or evidence-grading framework was applied, limiting systematic comparison of the methodological strength of individual studies. Quantitative synthesis was not performed because of substantial heterogeneity in biomarkers, analytical methods, sampling intervals, study populations, diagnostic definitions, and reported outcomes.

The underlying evidence base is itself highly heterogeneous. Studies differ substantially in population size and characteristics, stroke subtype and etiology, neurological severity, treatment exposure, inclusion of stroke mimics, and prevalence of cardiovascular or systemic comorbidities. The timing of blood collection represents an additional source of variability because individual biomarkers follow distinct temporal profiles after cerebral injury. Sampling is not consistently reported in relation to symptom onset, and serial measurements are used inconsistently, limiting direct comparison of biomarker kinetics across studies.

Analytical and preanalytical variability further complicates interpretation. Studies use different biological specimens, anticoagulants, processing protocols, assay platforms, detection limits, and analytical methodologies, resulting in considerable variation in reported concentrations and proposed decision thresholds. For many biomarkers, standardized reference intervals and clinically validated cut-off values remain unavailable. Circulating concentrations may also be influenced by age, renal or cardiovascular dysfunction, diabetes, infection, malignancy, chronic inflammatory or neurological disorders, and acute therapeutic interventions, reducing biomarker specificity for the cerebrovascular event itself.

Many candidate biomarkers and multimarker models have been evaluated predominantly in observational or single-center studies, frequently with limited sample sizes and internal validation only. Differences in outcome definitions, imaging protocols, follow-up intervals, and statistical approaches further restrict generalizability. Moreover, statistically significant associations or high discriminatory performance do not necessarily translate into clinical usefulness. Before routine implementation, biomarkers should demonstrate reproducibility across laboratories and populations, rapid availability, acceptable cost, clinically meaningful decision thresholds, and additional value beyond neurological assessment, routine laboratory testing, and neuroimaging. These methodological, analytical, and translational limitations remain major barriers to the incorporation of circulating biomarkers into routine emergency stroke pathways.

## 10. Future Directions and Clinical Perspectives

Future research on circulating biomarkers in acute stroke should increasingly focus on translation from exploratory associations to clinically applicable tools. Particular attention should be given to biomarkers that can provide rapid and reproducible information within the time-sensitive environment of prehospital and emergency care. Point-of-care platforms may facilitate this transition by allowing selected biomarkers to be measured close to the patient, potentially supporting early triage, imaging prioritization, and risk stratification. Their development should be accompanied by rigorous standardization of specimen collection, processing, storage, analytical procedures, and reporting of sampling time in relation to symptom onset.

Analytical harmonization will also be important because differences between laboratory platforms may limit the transferability of biomarker concentrations and decision thresholds across institutions. Future investigations should therefore prioritize reproducible assays, clearly defined analytical performance characteristics, and clinically meaningful cut-off values. Serial measurements may be useful for biomarkers with dynamic or delayed release kinetics, although their added clinical value should be weighed against additional costs, laboratory workload, and potential delays in emergency management.

Multimarker approaches may offer greater clinical relevance than isolated biomarkers by integrating complementary information on neuronal and glial injury, inflammation, coagulation, endothelial dysfunction, cardiac stress, and metabolic disturbances. However, increasingly complex panels are not necessarily more useful in clinical practice. Future models should favor a limited number of robust and rapidly measurable biomarkers that provide complementary information beyond established clinical assessment. Their integration with neurological scales and neuroimaging may provide a more comprehensive representation of both cerebral injury and systemic response.

AI and machine-learning methods may further support the integration of laboratory, clinical, and imaging data, particularly when relationships between variables are nonlinear or multidimensional. Future models should nevertheless remain interpretable, reproducible, and sufficiently transparent for clinical use. Model development should be followed by independent external validation, assessment of calibration, and comparison with established diagnostic and prognostic tools. Improvements in statistical performance should ultimately correspond to meaningful improvements in patient classification, triage, or clinical decision-making.

Prospective multicenter studies will be essential to determine whether biomarker-based strategies can be implemented across heterogeneous patient populations and different levels of stroke care. Future investigations should use predefined sampling protocols, clinically relevant endpoints, standardized analytical methods, and sufficiently diverse cohorts, including patients with important comorbidities and atypical presentations. Implementation should also consider turnaround time, laboratory availability, cost-effectiveness, staff training, interoperability with clinical information systems, and local stroke-network organization. Biomarker testing is most likely to become clinically useful when it provides rapid and actionable information while complementing, rather than delaying or replacing, established neurological assessment and neuroimaging.

## 11. Conclusions

Circulating biomarkers may complement clinical and imaging assessment in acute stroke by supporting diagnosis, subtype differentiation, severity assessment, and outcome prediction. Brain-specific and systemic biomarkers provide complementary information on cerebral injury and the biological response to stroke, although their interpretation remains influenced by sampling time, analytical variability, comorbidities, and stroke heterogeneity. Their greatest potential may lie in multimarker approaches integrated with neurological scales, neuroimaging, point-of-care testing, and AI-based models. Clinical implementation may require analytical standardization, validated decision thresholds, prospective multicenter studies, and demonstration of additional clinical value without delaying established diagnostic and therapeutic pathways.

## Figures and Tables

**Table 1:** Representative Clinical Evidence for Circulating Biomarkers in Acute Stroke.

Biomarker(s)	Clinical Use	Key Evidence
GFAP	Stroke subtype	Higher early levels in ICH; potential for ICH–ischemic stroke discrimination [54,55]
S100B, NSE, NfL, tau	Severity, prognosis	Associated with infarct burden, neurological severity, and outcome [57,58,59,60,61,62,63,64,65]
MMP-9	BBB injury, hemorrhagic transformation	Higher levels associated with hemorrhagic transformation and neurovascular injury [69,70,72]
CRP, IL-6	Inflammation, prognosis	Associated with neurological deterioration, recurrence, and poor outcome [81,82]
NLR, SII	Inflammation, prognosis	Associated with stroke severity and unfavorable outcome [86,87,88]
D-dimer, fibrinogen	Thrombosis, hemostasis	Associated with thrombotic burden, stroke mechanism, and outcome [89,90,91,92]
Troponin, BNP/NT-proBNP	Cardiac involvement	Reflect myocardial injury, cardiac stress, and cardioembolic mechanisms [93,94,95,96]
Lactate/metabolic markers	Metabolic stress, prognosis	May reflect systemic and metabolic stress associated with adverse outcome [97]

Note: Representative evidence is shown; the table is not intended to provide an exhaustive summary of all published studies. Abbreviations: BBB, blood–brain barrier; BNP, B-type natriuretic peptide; CRP, C-reactive protein; GFAP, glial fibrillary acidic protein; ICH, intracerebral hemorrhage; IL-6, interleukin-6; MMP-9, matrix metalloproteinase 9; NfL, neurofilament light chain; NLR, neutrophil-to-lymphocyte ratio; NSE, neuron-specific enolase; NT-proBNP, N-terminal pro-B-type natriuretic peptide; SII, systemic immune–inflammation index.

## Data Availability

No new data were created or analyzed in this study. Data sharing is not applicable to this article.

## Supplementary Materials

The following supporting information can be downloaded at: https://www.mdpi.com/article/10.3390/diagnostics16183053/s1, Table S1: Major Circulating Biomarkers in Acute Stroke: Biological Origin, Clinical Applications, and Translational Limitations. References cited in Table S1 correspond to the reference list of the main manuscript.
