# Supplementary material for: Clinical Utility of Circulating Biomarkers for Diagnosis and Prognosis of Acute Stroke in the Emergency Department: A Narrative Review from Current Evidence to Clinical Implementation
Source: Diagnostics (Basel). 2026 Sep 20;16(18):3053. doi: 10.3390/diagnostics16183053 (PMC13605812; doi:10.3390/diagnostics16183053)
Supplement: Supplementary file 1 [file diagnostics-16-03053-s001.zip › diagnostics-4557185-supplementary.pdf]

Table S1. Major Circulating Biomarkers in Acute Stroke: Biological Origin, Clinical Applications, and Translational Limitations

Biomarker / biomarker group	Principal biological origin	Temporal characteristics	Main potential clinical role	Major limitations / confounders	Representative references
**GFAP**	Astrocytic cytoskeleton; released after astrocytic injury and disruption of brain tissue integrity	Rapid and marked increase in ICH; generally slower increase after ischemic injury	Early differentiation of ICH from ischemic stroke; assessment of astrocytic injury and lesion burden	Time-dependent concentrations; lower sensitivity in small hemorrhages or very early ischemia; assay-dependent thresholds	[52,54,55]
**S100B**	Predominantly astrocytic protein, with additional extracerebral expression	Usually increases during the first hours after cerebral injury; less favorable hyperacute kinetics than GFAP	Assessment of brain injury, infarct burden, neurological severity, and functional outcome; potential stroke-subtype discrimination	Limited cerebral specificity; influenced by extracranial tissue injury and sampling time	[56–58]
**NSE**	Neuronal cytoplasm	Generally increases over several hours following neuronal injury	Assessment of neuronal damage, stroke severity, and outcome, particularly after reperfusion therapy	Hemolysis is a major preanalytical confounder; limited stroke and subtype specificity	[59]
**UCH-L1**	Neuronal cytoplasmic protein highly expressed in the central nervous system	Relatively early release after neuronal injury	Emerging marker of acute neuronal injury; potential contribution to multimarker diagnostic strategies	Limited independent validation; lack of standardized clinical cut-offs	[60]
**NfL**	Structural protein of large myelinated axons	Progressive and relatively delayed increase following axonal damage	Assessment of neuroaxonal injury, infarct burden, neurological severity, and functional outcome	Limited hyperacute utility; strongly influenced by age and chronic neurological or neurodegenerative disease	[61,62]
**Tau / brain-derived tau**	Axonal microtubule-associated neuronal protein	Generally increases progressively after neuronal and axonal injury	Assessment of neuronal injury, infarct volume, and longitudinal tissue damage	Delayed kinetics; conventional circulating tau is not completely brain-specific; assay heterogeneity	[63–65]
**MBP and other myelin-associated markers**	Myelin sheath and oligodendrocyte-related tissue injury	Predominantly delayed release as structural tissue injury evolves	Potential assessment of established myelin and white-matter damage	Limited hyperacute diagnostic value; relatively sparse modern validation data	[66,67]

**MMP-9**	Leukocytes, endothelial cells, and extracellular matrix remodeling within the neurovascular unit	Dynamic increase during acute inflammation, BBB disruption, and reperfusion	Assessment of neurovascular injury, BBB disruption, and risk of hemorrhagic transformation	Nonspecific systemic expression; influenced by inflammation, reperfusion therapy, and sampling time	[68–72]
**CRP and IL-6**	Systemic acute-phase and inflammatory responses involving hepatocytes, immune cells, and vascular endothelium	Dynamic increase during the acute inflammatory response; may persist for hours to days	Assessment of inflammatory burden, stroke severity, functional outcome, and recurrent vascular risk	Strongly influenced by infection, chronic inflammation, malignancy, and other systemic diseases	[57,78–83]
**Hematological inflammatory indices *(NLR, PLR, LMR, SII, SIRI)***	Derived from circulating leukocyte and platelet counts	May change rapidly during the acute systemic inflammatory response	Low-cost assessment of inflammatory burden, neurological deterioration, and functional prognosis	Nonspecific; influenced by infection, medications, physiological stress, hematological disorders, and baseline blood counts	[84–88]
**D-dimer**	Fibrin formation and degradation; systemic fibrinolytic activity	May increase early in thromboembolic stroke; concentrations vary with thrombotic burden and etiology	Identification of thromboembolic mechanisms and potential LVO; adjunctive etiological and prognostic assessment	Low specificity; strongly influenced by age, malignancy, infection, venous thromboembolism, trauma, and systemic coagulation activation	[89–91]
**Fibrinogen and other coagulation markers**	Hepatic coagulation proteins and systemic hemostatic pathways	Acute concentrations reflect both coagulation and inflammatory activation	Assessment of thrombotic state, vascular risk, and functional prognosis	Influenced by systemic inflammation, liver function, anticoagulant treatment, and acute-phase responses	[89,92]
**Cardiac troponins and natriuretic peptides**	Cardiomyocyte injury and myocardial wall stress	May be elevated at admission or change dynamically during the acute phase	Detection of concomitant myocardial injury, stroke–heart syndrome, and possible cardioembolic mechanisms; prognostic assessment	Influenced by acute coronary syndromes, renal dysfunction, atrial fibrillation, heart failure, and structural cardiac disease	[25,93–96]
**Lactate and metabolic/oxidative stress markers**	Anaerobic metabolism, systemic metabolic stress, and oxidative pathways	Often dynamic and strongly dependent on systemic and cerebral metabolic conditions	Exploratory assessment of disease severity, metabolic stress, and mortality risk	Low cerebral specificity; affected by seizures, systemic hypoperfusion, infection, hepatic dysfunction, and other critical illnesses	[44–49,97]

**miRNAs and extracellular vesicle-associated biomarkers**	Neurons, glia, endothelial cells, and immune cells involved in intercellular signaling	Highly molecule-specific and dependent on release, transport, and clearance mechanisms	Emerging diagnostic and prognostic signatures; potential incorporation into multimarker panels	Lack of standardized isolation, normalization, analytical platforms, and validated decision thresholds	[11,53,74,75]
**Proteomic and metabolomic signatures**	Integrated molecular responses involving neuronal, vascular, inflammatory, and metabolic pathways	Highly analyte- and pathway-dependent	Discovery of multianalyte diagnostic and prognostic signatures and potential integration with computational models	High dimensionality, batch effects, platform dependence, analytical complexity, and limited external validation	[12,77]

Note: Temporal characteristics are presented qualitatively because biomarker kinetics vary according to stroke subtype, lesion volume, reperfusion status, sampling interval, and analytical platform. Abbreviations: BBB, blood–brain barrier; CRP, C-reactive protein; GFAP, glial fibrillary acidic protein; ICH, intracerebral hemorrhage; IL-6, interleukin 6; LMR, lymphocyte-to-monocyte ratio; LVO, large-vessel occlusion; MBP, myelin basic protein; miRNA, microRNA; MMP-9, matrix metalloproteinase 9; NfL, neurofilament light chain; NLR, neutrophil-to-lymphocyte ratio; NSE, neuron-specific enolase; PLR, platelet-to-lymphocyte ratio; SII, systemic immune-inflammation index; SIRI, systemic inflammation response index; UCH-L1, ubiquitin C-terminal hydrolase L1.
